# Strong constitutive NF-κB signaling in B cells drives SLL/CLL-like lymphomagenesis and overcomes microenvironmental dependencies

**DOI:** 10.1038/s41375-025-02844-8

**Published:** 2026-01-16

**Authors:** Valeria Soberón, Lena Osswald, Andrew Moore, Dominika Sosnowska, Gene Swinerd, Jingyu Chen, Seren Baygün, Carina Diehl, Gönül Seyhan, Laura Kraus, Vanessa Gölling, Ricarda Trapp, Thomas J. O’Neill, Sabrina Bortoluzzi, Daniel Kovacs, Tim Ammon, Pankaj Singroul, Yuliia Hubarzhevska, Rupert Öllinger, Sebastian Mueller, Olga Baranov, Piero Giansanti, Felix Gillhuber, Sonja Grath, Oliver Weigert, Andreas Rosenwald, Yoshiteru Sasaki, Klaus Rajewsky, Katja Steiger, Florian Bassermann, Roland Rad, Daniel Krappmann, Ingo Ringshausen, Marc Schmidt-Supprian

**Affiliations:** 1 Institute for Experimental Hematology, School of Medicine and Health, Technical University of Munich, Munich, Germany; 2 Center for Translational Cancer Research (TranslaTUM), School of Medicine and Health, Technical University of Munich, Munich, Germany; 3 German Cancer Consortium (DKTK), Heidelberg, Germany; 4 Max-Planck Institute of Biochemistry, Planegg, Germany; 5 Department of Medicine III, School of Medicine and Health, Technical University of Munich, Munich, Germany; 6 Department of Haematology, University of Cambridge, Cambridge, UK; 7 Cancer Institute, University College London, London, UK; 8 Department of Biochemistry and Molecular Biology, West China School of Basic Medical Sciences & Forensic Medicine, Sichuan University, Chengdu, PR China; 9 Research Unit Signaling and Translation, Molecular Targets and Therapeutics Center, Helmholtz Zentrum München-German Research Center for Environmental Health, Neuherberg, Germany; 10 Institute of Molecular Oncology and Functional Genomics, School of Medicine and Health, Technical University of Munich, Munich, Germany; 11 Bavarian Center for Biomolecular Mass Spectrometry at Klinikum rechts der Isar, School of Medicine and Health, Technical University of Munich, Munich, Germany; 12 Division of Evolutionary Biology, Faculty of Biology, Ludwig-Maximilians-Universität (LMU) München, Planegg-Martinsried, Germany; 13 Laboratory for Experimental Leukemia and Lymphoma Research (ELLF), Faculty of Medicine, Department of Medicine III, Ludwig-Maximilians-University, Munich, Germany; 14 Institute of Pathology, University of Würzburg, Würzburg, Germany; 15 Program in Cellular and Molecular Medicine, Children’s Hospital, and Immune Disease Institute, Harvard Medical School, Boston, MA USA; 16 Division of Immunology, Faculty of Medicine, Tohoku Medical and Pharmaceutical University, Sendai, Japan; 17 Immune Regulation and Cancer, Max-Delbrück-Center for Molecular Medicine in the Helmholtz Association (MDC), Berlin, Germany; 18 Institute of Pathology, School of Medicine and Health, Technical University of Munich, Munich, Germany; 19 Bavarian Cancer Research Center (BZKF), Munich, Germany

**Keywords:** Chronic lymphocytic leukaemia, B-cell lymphoma, Preclinical research, Lymphoproliferative disorders, B-1 cells

## Abstract

Aberrant activation of NF-κB transcription factors is a hallmark of human lymphomas. Most lymphoma-intrinsic as well as microenvironment-induced NF-κB activation occurs upstream of the key kinase IKK2, therefore affecting additional pathways. Here, we show that canonical NF-κB signaling in mouse B cells, induced through the expression of one or two copies of a constitutively active IKK2 variant, dose-dependently drives lymphomagenesis. The observed phenotype and stereotypic B cell receptor clonality resemble human small lymphocytic lymphoma (SLL) and chronic lymphocytic leukemia (CLL). Stronger IKK2 signaling drives early B1a cell expansion and uniform SLL/CLL-like lymphomagenesis, while intermediate signals cause more heterogeneous malignancies. Mechanistically, constitutive IKK2 signals provide a profound cell-intrinsic competitive advantage to B1a cells and dose-dependently synergize with TCL1 overexpression in driving aggressive CLL. Further, strong constitutive NF-κB activation overcomes critical microenvironmental dependencies of TCL1-driven lymphomas. Our findings establish canonical NF-κB as an oncogenic driver in lymphoma and reveal reduced microenvironment dependency as a key NF-κB-mediated mechanism, thus highlighting its therapeutic relevance.

## Introduction

NF-κB/Rel, a transcription factor family with critical functions in physiology and disease, regulates proliferation, differentiation, survival and functions of immune cells [1, 2]. Physiological NF-κB activation occurs mainly via two signaling cascades termed canonical and alternative pathway. The canonical pathway is activated by various upstream signals leading to activation of the IκB-kinase (IKK) complex composed of the catalytic subunits IKK1/IKKα and IKK2/IKKβ and the regulatory NF-κB essential modulator (NEMO). Activated IKK phosphorylates inhibitory IκB proteins thereby inducing their ubiquitin-mediated proteolytic degradation, which results in the release and nuclear translocation of hetero- and homodimers of mostly c-Rel, RelA and p50, followed by reprogramming of gene expression. On the other hand, alternative NF-κB signaling involves activation of IKK1 by NF-κB-inducing kinase (NIK), leading to processing of NFΚB2/p100 to p52 and activation of NF-κB complexes consisting mostly of p52 and RelB [1, 2].

NF-κB proteins were discovered in B cells [3] and they play key roles in B cell development, survival and functions. Various receptors that mediate key functions in B cells activate canonical NF-κB signaling, including the B cell receptor (BCR), CD40 and Toll-like receptors (TLRs) [4]. We previously demonstrated that the IKK complex is not strictly required for the development of B cells in the bone marrow, but mediates the maintenance of mature peripheral B cells and the differentiation of λ-light chain expressing B cells, marginal zone B (MZB) cells as well as B1 cells [5–8]. Conversely, B cell-specific continuous activation of canonical NF-κB through expression of a constitutively active IKK2 variant (IKK2ca) increased the numbers of mature B cells, especially MZB cells, which were expanded over five-fold [8]. IKK2ca-mediated NF-κB activation released B cells from their dependence on BAFFR-mediated maintenance signals and while it did not induce spontaneous B cell proliferation, it strongly enhanced B cell expansion after BCR cross-linking [8]. Furthermore, expression of IKK2ca starting in germinal center B (GCB) cells caused premature termination of GC reactions and plasma cell hyperplasia in aged mice [9]. Fitting to their crucial roles in B cell survival and proliferation, aberrant activation of NF-κB transcription factors is a hallmark of human lymphomas, the most prevalent hematologic malignancies [10, 11]. This occurs mostly through mutations in proteins regulating signaling upstream of the IKK complex, and therefore, additional pathways are affected as well. Another important source of NF-κB activity in lymphomas is their tumor microenvironment, which induces high expression of NF-κB target genes also in entities with less prevalent NF-κB-activating genetic lesions, including multiple myeloma (MM) [10, 12] and B-cell chronic lymphocytic leukemia (CLL) [13–16]. Genetic alterations that exclusively increase or amplify canonical NF-κB signals include loss/inactivation of IκB proteins, gain of c-REL and activating mutations in IKK2 [11]. The latter is most prominent in splenic marginal zone B cell lymphoma [17], but was also detected in mediastinal B cell lymphoma (PMBCL) [18], diffuse large B cell lymphoma (DLBCL) [11] and MM [19]. Functional studies in mice revealed that IKK2ca-mediated NF-κB activation strongly promotes lymphomagenesis driven by DLBCL-associated loss of BLIMP1 [9], gain of PI3K activity [20], gain of c-MYC [21] as well as MM-associated gain of CCDN1 and MMSET [22].

To resolve the open question of the potential of different strengths of constitutive NF-κB signaling as a founding oncogenic driver in B cells, we analyzed cohorts of mice with B cell-specific expression of one or two copies of the IKK2ca (IKK2ca/ca) transgene over time. IKK2ca/ca expression induced a progressive expansion of innate-like B1a cells, culminating in a small lymphocytic lymphoma (SLL)/CLL-like disease with a course and lethality comparable to Eµ-hTCL1^tg^ (TCL1^tg^) mice [23], the most prominent preclinical CLL mouse model. IKK2ca-mediated NF-κB activation dose-dependently accelerated lymphomagenesis when paired with transgenic TCL1 expression. This correlated with a pronounced cell-intrinsic competitive advantage of B1a cells expressing constitutive NF-κB and the striking relief of IKK2ca/ca expressing TCL1^tg^ CLL cells from critical microenvironmental dependencies.

## Methods

### Mice

The mouse strains B6.129P2(C)-*Cd19*^*tm1(cre)Cgn*^/J (CD19Cre) [24], B6.129P2(Cg)-*Ighg1*^*tm1(cre)Cgn*^/J (Cγ1Cre) [25], B6.129P2-Aicda^tm1(cre)Mnz^/J (AIDCre) [26], B6.Cg-Gt(ROSA)26Sor^tm4(Ikbkb)Rsky/^J (IKK2ca) [8], Gt(ROSA)26Sor^tm1(CAG-CARΔ1(StopF)^ (CAR) [27], B6.Cg-Gt(ROSA)26Sor^tm9(CAG-tdTomato)Hze^/J (tdTomato) [28], Eµ-hTCL1tg [23] and Prkcb^tm1Tara^ (PKC-β^−/−^) [29] were published and provided by the authors. All mouse strains used for this study were generated or backcrossed to a C57BL/6 background. All mice were bred and kept in specific pathogen-free (SPF) or specific and opportunistic pathogen free (SOPF) conditions according to the guidelines of and approval by the Government of Upper Bavaria (55.2-1-54-2532-95-09, 55.2-1-54-2532-234-2015, 55-2-2532.Vet_02-21-115, 55-2-2532.Vet_02-23-111) and the European Union and the Animals Act 1986 Amendment Regulations 2012 following ethical review by the University of Cambridge Animal Welfare and Ethical Review Body (AWERB-PPL number P846C00DB). Mice were housed at the mouse facilities of the Max-Planck Institute of Biochemistry in Munich, the Centre for Preclinical Research of the MRI (Zentrum für Präklinisches Forschung, ZPF) in Munich, Charles River Calco in Italy, and the Ann Mclaren Building in Cambridge, UK.

All mice were genotyped by PCR analysis from DNA extracted from tail or ear biopsies. The Primer sequences are shown in Table S1A. Sample size was not predetermined by statistical calculation but was chosen to ensure adequate representation of biological variability. Randomization was not used because experimental groups were defined by genotype. The investigators were not blinded during data collection or analysis.

### Blood and organ processing

Blood samples were collected at indicated time points by puncturing the vena facialis. Samples were further analyzed with an animal blood cell counter (scil) and by flow cytometry after erythrocyte lysis with ACK lysis buffer (Gibco).

Mice were sacrificed at indicated time points and organs were harvested and processed for further analysis. Briefly, spleen and lymph nodes were dissociated between two microscopic glass slides, resuspended in MACS buffer (2 µM EDTA, 1% FBS, 0.25% BSA, 1% Pen/Strep in PBS) and filtered before further analysis. Bones (femur and tibia) were crushed in B cell media or MACS buffer with a mortar to obtain bone marrow. Peritoneal cavity cells were obtained by flushing the peritoneum with 5 ml of ice-cold MACS buffer, Gey’s solution was used to lyse erythrocytes from spleen and bone marrow.

### Flow cytometry

One to five million cells per sample were stained in 96 well V-bottom plates. Cells were stained with the CD16/CD32 antibody to prevent unspecific binding to Fc receptors. Cell viability was assessed by staining cells with 7-AAD/AnnexinV (eBioscience), or the fixable LIVE/DEAD Near-IR (Invitrogen) staining or iFluor^(R)^ 840 melamide (AAT Bioquest). Samples were stained with extracellular antibodies in FACS buffer. For intracellular stainings, cells were fixed in 2% PFA followed by methanol permeabilization, processed using the Foxp3/Transcription Factor Set (eBioscience), or fixed in Roti-histofix^(R)^ (Roth) followed by the FoxP3/Transcription Factor set. For intracellular flow cytometry of phosphorylated proteins, cells were stained using the BD Pharmingen Phosflow kit, following manufacturer’s instructions. Flow cytometry antibodies are shown in Table S1D. Cells were acquired in BD FACS Canto^TM^ II System (BD Bioscience, Cat. No. 338962), BD FACS Canto^TM^ (BD Bioscience, Cat. No. 657338), Cytoflex-S, Cytoflex-LX (Beckman Coulter Life Sciences) flow cytometers. Flow data were analyzed using FlowJo V9 and 10 (BD). Doublets and debris were excluded through sequential gating using FSC-A vs. FSC-H and SSC-A vs. SSC-Width. Subsequently, gating on live cells was performed to exclude dead cells from the analysis. Gating strategies are indicated in Table S2 and/or in the Figure legends.

### Flow-cytometry-based purification of cells

For bulk RNA sequencing, target cells were first stained with the anti-mouse CD16/CD32 monoclonal antibody (eBioscience) on ice for 30 min to prevent nonspecific antibody binding. Cells were then stained extracellularly with antibodies against CD19 (Clone eBio1D3), B220 (clone RA3-6B2), IgM (clone II/41), IgD (clone 11-26c/11-26) and CD5 (Clone 53-7.3). For exclusion of dead cells, either live-dead dye 7-AAD (eBioscience) or the near-IR dead cell staining kit (Invitrogen) was used. Cells were then resuspended in FACS Buffer, filtered through a 35 µm nylon mesh (Corning) and sorted for single, living B1a cells (CD5^+^ B220^low^ CD19^+^) with BD FACS Aria™ II, BD FACS Aria™ III or BD FACS Aria™ Fusion.

One thousand B1a splenic and peritoneal cells from CD19Cre control, CD19Cre IKK2ca, and CD19Cre IKK2ca/ca were sorted into each well of a 96-well PCR plate pre-filled with 5 µl buffer TCL (Qiagen). When possible, two wells (technical replicates) were sorted for each population. The plates were then sealed, centrifuged and stored at −80 °C. For tumorigenic B1a-like cells from CD19Cre TCL1^tg^, CD19Cre IKK2ca TCL1^tg^ and CD19Cre IKK2ca/ca TCL1^tg^ the maximum number of cells possible was sorted per sample. Tubes were centrifuged, supernatant was carefully removed, and samples were immediately lysed in 350 µl RLT lysis buffer and stored at −80 °C.

### Magnetic-based purification of cells

For comparative genomic hybridization (CGH) array analysis, bulk RNA sequencing of tumors, and transplantation experiments, B1a-like cells were purified from lymphomas either freshly or by thawing viable cells stored in 10% DMSO (GibCo) in FBS or cell freezing media CELLBANKER2^Ⓡ^ (Amsbio). Single cell suspensions were processed in MACS buffer (2 µM EDTA, 1% FBS, 0.25% BSA, 1% Pen/Strep in PBS). Percoll gradient centrifugation was selectively used to remove dead cells from a single cell suspension. CD5^+^ lymphoma cells were manually magnetically purified by staining cells with a cocktail of biotinylated antibodies (Gr1^+^ (RB6-8C5, BioLegend), Ter119^+^ (clone Ter119, eBioscience), CD3^+^(clone 145-2C11, BD), F4/80^+^ (clone CI:A3-1, AbD Serotec), CD11c^+^ (clone N418, eBioscience) and IL7R^+^ (CD127, clone A7R34, eBioscience)) followed by depletion of the stained cells using anti-Biotin magnetic beads (Miltenyi) and the LS-columns (Miltenyi), according to the manufacturer’s instructions. Negative enrichment purity of the population of interest was validated by flow cytometry, comparing before and after fractions. In samples with broad B220 expression, an additional round of depletion of B220^high^ expressing B cells was performed by using a B220-biotinylated antibody (clone RA3-6B2, eBioscience). Only samples with >95% purity post-enrichment were used in later applications.

### In vitro proliferation assay

FACS-purified or MACS-enriched B1a-like cells (CD19^+^ B220^low^ CD5^+^ and eGFP^+^ for the samples expressing IKK2ca) were pre-labeled with the eBioscience Cell Proliferation Dye eFluorTM 450 (Thermo Fisher Scientific, Cat. No. 65-0842-85) following manufacturer’s instructions. Ex vivo cell proliferation was evaluated under resting conditions by flow cytometry. Cell proliferation was assessed using the FlowJo Proliferation analysis Package (BD).

### Comparative genomic hybridization (CGH) array

Suspected tumor genomic DNA was isolated from spleens from burdened mice, while reference genomic DNA was isolated from tail or ear biopsies. Moreover, DNA was isolated from the cell lines TR28 and TR50, that were derived from CD19Cre IKK2ca/ca lymphomas upon serial transfers into recipient mice (Figs. 2H and S2H). The CGH array was performed according to the manufacturer’s protocol (Agilent Technologies) and scanned with the DNA microarray scanner G2505C (Agilent Technologies). Copy number segmentation was performed on the CGH probe data using the circular binary segmentation (CBS) algorithm from the PSCBS package (version 0.66.0) in R (version 4.3.2). The segments were disjoined and overlapped for each sample to be visualized as a copy ratio heatmap. Only regions with a log2 copy ratio below 0.2 or above 0.2 and a width larger than 10.000 bp were considered. Z-score normalization was performed on the data.

### IgH VDJ rearrangement by PCR-cloning-sequencing, Southern blotting, and next-generation sequencing

#### PCR-cloning-sequencing

To assess clonality of potential lymphomas, the rearrangement of the immunoglobulin heavy chain (VDJ) was analyzed by amplification, cloning and sequencing. Three different PCRs were performed on genomic DNA extracted from purified CD5^+^ B cells, which were isolated from lymphoproliferative tissue.

First, amplification of the IgH rearrangements for the different IGHJ gene segments (JH1-JH4) was performed to assess if a particular IGHJ gene segment was predominantly used compared to the other three, indicative of potential clonal expansion. For this first PCR, the following thermal cycler conditions were used: 98 °C for 10 min, 35 cycles of 98 °C for 10 s, 67 °C for 30 s, 72 °C for 30  s and 72 °C for 10 min. Amplicons were visualized by agarose electrophoresis, and an initial assessment for rearrangement into all IGHJ gene segments was made.

Rearrangements into Jh1 and Jh4, and rearrangements into Jh2 and Jh3 were further amplified in two separate PCR reactions, respectively. The different rearrangements were later cloned using Golden Gate cloning into specific destination vectors for the different JH rearrangements that recognize specific sequences in the 3’ end of the amplicons, termed catch sequence, for each rearrangement [30]. 10–90 colonies were picked per sample and sent for Sanger sequencing to Eurofin Genomics EU (Ebersberg). Analysis was performed with SeqMan Pro (DNASTAR V15.0). Sequences were exported and annotated using the IMGT/HightV-Quest v 1.9.3 tool (imgt.org), including the IGHV identity to germline. Sequences of all primers that were used for amplification and sequencing are shown in Table S1B.

#### Southern blotting

Southern blotting for lymphoma clonality was performed with StuI-digested genomic DNA from MACS-purified B cells or frozen tissues using a JH probe spanning the JH4 exon and part of the downstream intronic sequence [9, 31].

#### BCR next-generation sequencing for tumor clonality and clonal tumor load

MACS- or FACS-purified B1a cells from spleens from sick mice were lysed in RLT buffer and their RNA extracted using the RNeasy Plus kit (Qiagen), following manufacturer’s instructions. Eluted RNA was treated with DNase to remove residual gDNA. First, messenger RNA for the IgM BCR sequence was reverse-transcribed using a mu-specific primer (IgM reverse primer binding in the mu chain), followed by template switch. DNA glycosylase was used to remove residual template switch adapters. cDNA was purified using the Agencourt AMPure XP beads (Beckman Coulter). Then, the cDNA was amplified in three rounds [32]. Sequences of primers that were used for amplification are shown in Table S1C. The PCR product was purified using the AMPure XP beads and sequenced with the MiSeq instrument. Analysis was done using symmetric paired-end Illumina MiSeq sequencing, standard Illumina Nextera sequencing primers and MiSeq Reagent Kit v3.

#### Bulk 3’ RNA sequencing

B1a cells were purified as described above. The bulk 3’-sequencing of poly(A)-RNA library preparation was done as described previously [33]. Briefly, frozen cell lysates from sorted B1a cells were thawed and RNA was isolated using either Agencourt AMPure XP magnetic beads when 1000 cells were sorted per well or RNeasy kit (Qiagen) for large amounts of cells. Barcoded cDNA of each sample was generated with a Maxima RT polymerase (Thermo Fisher) using oligo-dT primer containing barcodes, unique molecular identifiers (UMIs) and an adapter. 5’-Ends of the cDNAs were extended by a template switch oligo (TSO) and full-length cDNA was amplified with primers binding to the TSO-site and the adapter. cDNA from all samples was pooled and amplified with KAPA HiFi ReadyMix (KAPA Biosystems). To obtain sequencing libraries, samples were tagmented and 3’ ends were amplified with the Nextera XT Kit (Illumina). The library was sequenced on a NextSeq 500 (Illumina) with 16 cycles for the barcodes and UMIs in read1 and 51 cycles for the cDNA in read2. Data were processed using the published Drop-seq pipeline (v1.12) to generate sample- and gene-wise UMI tables [34]. STAR 2.7.5b was used for alignment to the reference genome (GRCm38). Transcript and gene definitions were used according to the GENCODE version M25.

Differential gene expression analysis was done with the DESeq2 package in R (version 4.3.2), which employs a negative binomial distribution model to identify differentially expressed genes between conditions (here: genotypes). A gene was considered significantly differentially expressed between two groups if the adjusted *p* value was <0.05 and the absolute log2 fold change was > 0.58 (corresponding to a 1.5 fold change). Gene set enrichment analysis (GSEA) was done with the fgsea package or the cluster Profiler package (version 4.14.3) [35] in R (version 4.4.1) using the GSEA and gseGO functions. Genes were ranked according to “stat” and a baseMean threshold of >2 was applied before running GSEA. The gene signatures used for GSEA are shown in Table S3A, G, KEGG gene signatures were obtained from the KEGG PATHWAY database. As indicated, the Zhao gene signature was supplemented by 10 additional targets: Birc3, Ccl22, Cd40, Cd80, Fcer2, Ikbke, Nfkb2, Rela, Relb, and Tnfrsf9.

### Adoptive transplantation experiments

#### Transfer of IKK2ca- and IKK2ca/ca-expressing B cells into wild-type and Rag2^−/−^ cγ-chain^−/−^ recipients

Ten to twenty-five million MACS-purified B cells from the spleen or peritoneal cavity of aged CD19Cre IKK2ca and CD19Cre IKK2ca/ca were intravenously or intraperitoneally injected into wild-type or Rag2^−/−^ cγ-chain^−/−^ mice. Mice were closely monitored after transplantation and euthanized upon signs of distress or enlarged spleens.

#### Transplantation of TCL1^tg^ CLL cells into PKCβ-proficient and PKCβ-deficient mice

Twenty million MACS-purified splenic B or B1a-like cells from lymphoma-bearing CD19Cre TCL1^tg^, CD19Cre IKK2ca TCL1^tg^, and CD19Cre IKK2ca/ca TCL1^tg^ mice were injected intraperitoneally into age-matched control PKC-β proficient (wild-type or PKC-β^+/−^) and PKC-β deficient (PKC-β^−/−^) recipient mice. Mice were closely monitored after transplantation. Mice were initially bled 1 week after transplant and then every 2 weeks for a period of 6 months to monitor the apparition of transplanted B1a-like cells in peripheral blood. At detection of indications of disease burden (more than 50% B1a-like cells in PB, splenomegaly and/or anemia), animals were euthanized, and blood and organs were collected for further analysis. Same-sex littermates were divided into control or experimental groups to minimize variability; no formal randomization procedure was applied. The investigators were not blinded to the group allocation during the experiments and analysis.

#### Western blotting

B cells were purified from spleens by MACS-depletion of non-B cells using the pan B cell isolation kit (Miltenyi), cell pellets were stored at −80 °C until further analysis. Cell pellets were lysed in co-IP lysis buffer (25 mM HEPES pH 7.5, 150 mM NaCl, 0.2% NP-40, 10% glycerol, 1 mM DTT, 10 mM NaF, 8 mM -glycerophosphate, 300 μM sodium vanadate and protease inhibitor cocktail mix (Roche)) for 20 min at 4 °C. Cellular debris was removed by high-speed centrifugation (20,000 *g*, 15 min, 4 °C), the supernatants were mixed with 4x SDS loading buffer (ROTI-load, Roth) and samples were boiled at 95 °C for 5 min. The lysates were separated by SDS-PAGE electrophoresis at 90-120 V followed by semi-dry blotting onto PVDF membranes. Membranes were blocked with 5% milk in PBS-Tween (PBS-T) for 1 h at room temperature, then incubated with primary antibodies (Table S1E) in PBS-T plus 2.5% BSA or milk at 4 °C overnight. Membranes were washed 3× in PBS-T for ten minutes each and then incubated with HRP-conjugated secondary antibodies (Table S1E) in PBS-T + 1.25% milk or BSA for 1 h at room temperature. Following another three washes, protein bands were visualized using the LumiGLO reagent kit (Cell Signaling). Western blot quantification was done using the AzureSpot Pro image analysis software (version 1.41) from Azure Biosystems.

#### Histological and immunohistochemical analysis

Murine organs were fixed in 4% paraformaldehyde for 48 h and subsequently embedded in paraffin. Tissue sections were stained with Hematoxylin & Eosin (Thermo Scientific) for morphological analysis or stained immunohistochemically using the following antibodies: CD45R (B220) (Clone RA3-6B2, BD, 550286, 1:50), CD5 (Clone E6N9S, Cell Signaling, #10084, 1:200), Ki67 (Clone SP6, Abcam, ab16667, 1:50), CD138 (Clone EPR6454, Αbcam, ab128936, 1:8000) and IRF4 (polyclonal, Santa Cruz, sc-6059, 1:1000). All stainings were performed on a Leica Bond Rxm after deparaffinization and pretreatment with H1 (corresponding to citrate pH6) for CD45R(B220), CD5, Ki67, and IRF4 or with H2 (corresponding to EDTA pH8) for CD138 for 30 min. The primary antibody binding was detected with a Leica polymer refine detection kit. For CD45R(B220) a rabbit anti rat (Vector, AI-4001, 1:400) and for IRF4 a rabbit anti-goat (Vector, AI-5000, 1:1000) secondary antibody was used. All slides were digitalized using a Leica Aperio AT2 scanning system. Histopathological assessment was done by two experienced pathologists (A.R. and K.S.) and all neoplasms were classified according to the consensus classification of lymphoid neoplasms in mice [36] and the classification of non-proliferative and proliferative lesions of the rat and mouse hematolymphoid system by INHAND (International Harmonization of Nomenclature and Diagnostic Criteria for Lesions in Rat and Mice) [37]. Proliferative activity in neoplastic cells was assessed by an experienced board-certified pathologist (K.S.). The percentage of Ki-67 positive expanding B cells (CD19 positive) was assessed using an Olympus BX53 brightfield microscope.

#### Patient-derived CLL cells

Primary CLL cells were isolated from the peripheral blood of CLL patients by Ficoll gradient centrifugation and frozen until further analysis. All patients have provided informed consent in accordance with the Declaration of Helsinki (REC reference number: 25/YH/0120), IRAS Project ID 355220).

#### Secretome analysis of primary CLL cells

Primary CLL cells from 20 different patients were co-cultured in serum-free IMDM medium with either EL08 stroma cells or YK6 follicular dendritic cells overexpressing CD40L and interleukin 21 (“FDC CD40L IL21”) [38]. Flow cytometry confirmed a percentage of >95% of CD19^+^ CD5^+^ cells within the leukocyte compartment. Supernatants were collected after 6 h, centrifuged, filtered and processed by acetone precipitation before being analyzed by high-sensitivity mass-spectrometry-based proteomics. Secretomes were denaturated, reduced, and alkylated with 1% sodium deoxycholate (SDC), 10 mM tris(2-carboxyethyl)phosphinehydrochloride (TCEP), 40 mM chloroacetamide, and 150 mM Tris-HCl, pH 8.5 (all Sigma), for 10 min at 95 °C and 1000 rpm. Proteins were digested overnight under gentle agitation (700 rpm) at 37 °C with trypsin (Thermo Fisher Scientific) with an enzyme:substrate ratio of 1:50. SDC was precipitated by acidification to 5% of formic acid (FA). Prior to mass spectrometry analysis, samples were desalted by the AssayMAP Bravo Platform (Agilent) using RP-S cartridges (5 μL bed volume, Agilent) and the standard peptide cleanup v3.0 protocol.

LC-MS/MS measurements were performed using a Vanquish Neo UHPLC coupled to the Orbitrap Exploris 480 mass spectrometer (Thermo Fisher Scientific). Peptides were delivered to a trap column (75 μm i.d. × 5 cm, packed in-house with 5 μm of Reprosil C18 beads; Dr. Maisch) using 0.1% formic acid at a flow rate of 5 μL/min. Subsequently, peptides were transferred to an analytical column (75 μm i.d. × 43 cm, packed in-house with 1.9 μm Reprosil C18 beads, Dr. Maisch) at a flow rate of 300 nL/min and chromatographically separated using a 171 min linear gradient of solvent B (0.1% formic acid, 3% DMSO in ACN) and solvent A (0.1% formic acid, 3% DMSO in water). The total measurement time for each sample was 180 min.

A data-independent acquisition (DIA) method with a label-free quantification strategy was used to determine the relative abundance of secreted proteins. DIA was performed with one full MS scan followed by 46 MS/MS covering precursor masses from 360 m/z to 1300 m/z with variable isolation windows. The full MS events were recorded with an AGC of 300%, a maxIT of 50 ms and a resolution of 120,000. MS/MS spectra were acquired with an NCE of 30% using an AGC of 3000%, a maxIT of 54 ms, and a resolution of 30,000.

DIA-NN 1.8.1 [39] was used to analyze DIA data against an in-silico-generated library from the UniProtKB Human database (UP000005640, containing canonical and isoform sequences, downloaded on 10/2023). The default settings were kept, while the charge state was set to 2–4, and the precursor’s m/z range was restricted from 360 to 1300 m/z. Identifications were filtered for a maximum of 1% FDR at precursor and global protein levels. The R package *iq* was used to calculate protein MaxLFQ values.

#### Statistics

Statistical analyses were done with GraphPad Prism (v10.4). Briefly, the normal distribution of the data was assessed using the Shapiro-Wilk test. If all groups were normally distributed, one-way ANOVA followed by Tukey’s multiple comparisons test or a one-tailed t-test was performed. If one or more groups did not follow normal distribution, Kruskal-Wallis test followed by Dunn’s multiple comparisons test or a one-tailed Mann-Whitney test was performed. Detailed information on all statistical analyses is shown in Table S2. No statistical methods were used to predetermine sample size.

## Results

### Constitutive IKK2 signals dose-dependently induce NF-κB target gene expression and mediate differential effects on B cell subset expansion

**Fig. 1:**
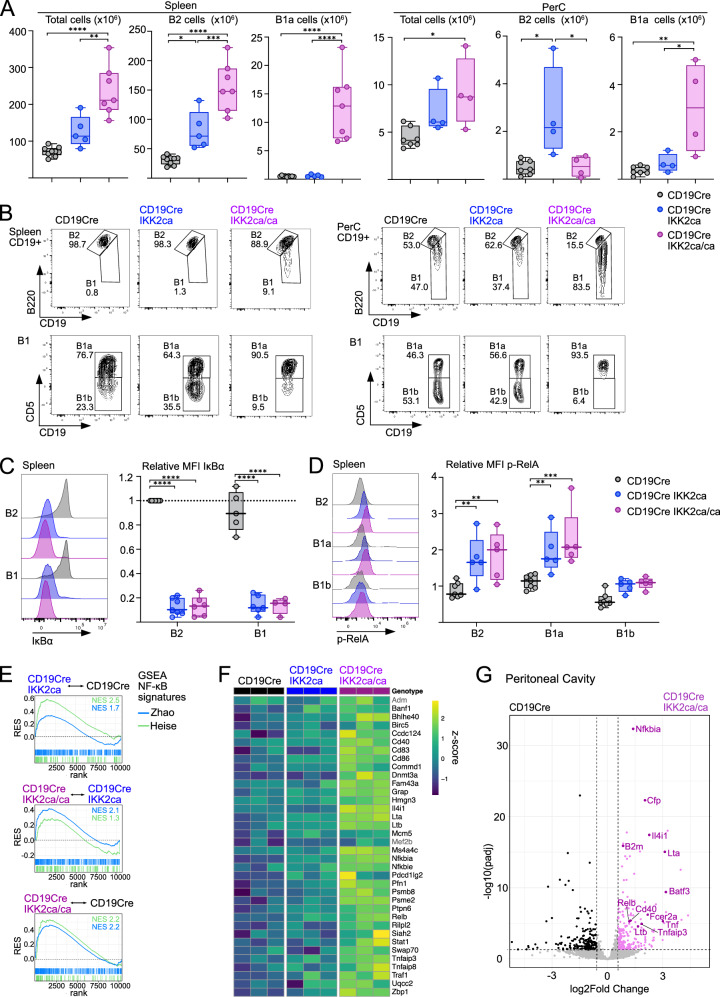
Constitutive IKK2 signals induce dose-dependent induction of NF-κB target genes and B cell expansion in young mice. **A**, **B** Ex vivo analysis of spleen and peritoneal cavity (PerC) of 3-month-old CD19Cre IKK2ca and CD19Cre IKK2ca/ca mice compared to CD19Cre control mice. **A** Total cell numbers, absolute B2 cell and B1a cell numbers in spleen and peritoneal cavity. **B** Representative flow cytometry plots depicting B cell subpopulations in spleen and peritoneal cavity (PerC), gated on CD19^+^ B cells (top) and CD19^hi^ B220^low^ B1 cells (bottom). **C** Intracellular flow cytometry analysis of IκBα degradation in unstimulated freshly isolated or previously frozen splenocytes from young mice (3–4.5 months old). Representative histograms and relative mean fluorescent intensities (MFI) are shown. MFI was normalized to B2 cells in CD19Cre control samples (4 independent experiments). **D** Intracellular phos-flow cytometry analysis of phosphorylated RelA (p-RelA) levels in unstimulated ex vivo isolated splenocytes from young mice (3–4.5 months old). Representative histograms and relative MFI are shown. MFI was normalized to T cells for each sample (3 independent experiments). **E**–**G** Bulk RNA sequencing analysis of FACS-purified B1a cells isolated from the peritoneal cavity of 3-month-old mice. **E** Gene set enrichment analysis (GSEA) of published NF-κB signatures in peritoneal B1a cells of indicated mouse genotype comparisons. **F** Heatmap of NF-κB target genes that are significantly upregulated in peritoneal B1a cells of CD19Cre IKK2ca/ca mice compared to CD19Cre mice (|log2FC| > 0.58, adjusted *p* value < 0.05). Additional genes that are only significantly upregulated in splenic B1a cells (see Fig. S1G) are depicted in gray (*Adm*, *Mef2b*). **G** Volcano plot showing differentially expressed genes in B1a cells from CD19Cre IKK2ca/ca mice compared to CD19Cre control mice. *p ≤ 0.05, **p ≤ 0.01, ***p ≤ 0.001, ****p ≤ 0.0001, one-way ANOVA followed by Tukey’s multiple comparison test or two-way ANOVA. B (CD19^+^), B2 (B220^hi^ CD19^+^), B1 (B220^low^ CD19^hi^), B1a (CD5^+^ B220^low^ CD19^hi^) and B1b (CD5^−^ B220^low^ CD19^hi^). *RES* running enrichment score, *NES* normalized enrichment score, *rank* rank in gene list (ranked by stat). Gene signatures used for GSEA are shown in Table S3A. GSEA statistics are shown in Table S3B, differentially expressed genes are shown in Table S4. See also Fig. S1. For more information on statistical analyses, see Table S2.

To investigate the effects of different levels of constitutive canonical NF-κB activation on B cells, we generated mice with B cell-specific expression of one or two copies of a Rosa26 knock-in IKK2ca allele [8] (R26-LSL-IKK2ca = IKK2ca), referred to as “CD19Cre IKK2ca” and “CD19Cre IKK2ca/ca” mice, respectively. A co-expressed eGFP serves as a surrogate marker for IKK2ca expression in our analyses. Already in 3-month-old mice, expression of one or two IKK2ca copies led to nearly linear increases in splenomegaly and splenic B2 cell hyperplasia, while T cell numbers were not altered (Figs. 1A and S1A). The most striking difference caused by the expression of a second IKK2ca copy was a prominent expansion of CD19^hi^ B220^low^ CD5^+^ B1a cells in spleen and peritoneal cavity, which in the latter seemed to come at the expense of B2 cells (Figs. 1A, B and S1B). To monitor NF-κB activation in B cell subsets, we investigated key pathway components by flow cytometry and Western blot. We detected an overall 4-fold and 8-fold increase in IKK2 expression (IKK2ca plus endogenous IKK2) in splenic IKK2ca- and IKK2ca/ca-expressing B cells, respectively (Fig. S1D). Our analyses further confirmed, as previously reported [8], massively reduced IκBα protein levels in B2 and B1 cells in the spleen and peritoneal cavity of CD19Cre IKK2ca mice, which were, however, not further reduced through the expression of an additional IKK2ca copy (Figs. 1C and S1C). Nearly complete absence of IκBα was also observed in Western blots of splenic B cells (Fig. S1D). Further, there was a trend for higher levels of phosphorylated RelA and increased protein levels of the NF-κB targets p100, RelB and IκBε in IKK2ca/ca- compared to IKK2ca-expressing B cells (Figs. 1D and S1C, D), indicating stronger NF-κB activation in the former cells.

To determine the effects of graded IKK2 signaling on gene expression, we FACS-purified splenic and peritoneal B1a cells, the most altered B cell subset, from young mice and performed bulk RNA sequencing. Gene set enrichment analysis (GSEA) of B cell-derived [40, 41], DLBCL-derived (Staudt, lymphoma project), CLL-derived [13] NF-κB signatures as well as NF-κB target genes obtained from the collecTRI database [42] showed an increase in NF-κB target gene expression in peritoneal B1a cells expressing one IKK2ca copy, which was significantly further enhanced by the expression of two copies (Figs. 1E and S1E, Table S3B). This is also reflected at the level of significantly elevated individual NF-κB target genes, including *Cd40*, *Cd86*, *Il4i1*, *Nfkbia*, *Nfkbie*, *Relb* and *Tnfaip3* as well as lymphotoxin α and β (*Lta* and *Ltb*) (Fig. 1F). High expression of *Nfkbia* transcripts in conjunction with almost complete absence of IκBα protein emphasizes the strong potency of IKK2ca to induce rapid degradation of re-synthesized IκBα and thus to induce constant canonical NF-κB signaling. Whereas expression of one copy of IKK2ca resulted in only minor gene expression changes in ex vivo isolated peritoneal B1a cells (17 upregulated, 12 downregulated genes), 223 genes were significantly upregulated in IKK2ca/ca- versus control B1a cells, amongst them several genes implicated in lymphoma biology, including *B2m* [43], *Il4i1* [44], *Tnf* [45], *Lta* [46], and *Batf3* [47] (Fig. 1G and Table S4A–C). Similar trends were observed in splenic B1a cells (Fig. S1F–H and Table S4D–F).

Our analyses thus revealed that graded levels of IKK2ca expression led to proportional NF-κB activation as well as expansion of resting B2 cells in young mice, while strong constitutive NF-κB activation in addition caused an early expansion of B1a cells.

### Dose-dependent induction of lymphomagenesis by constitutive IKK2 signaling

**Fig. 2:**
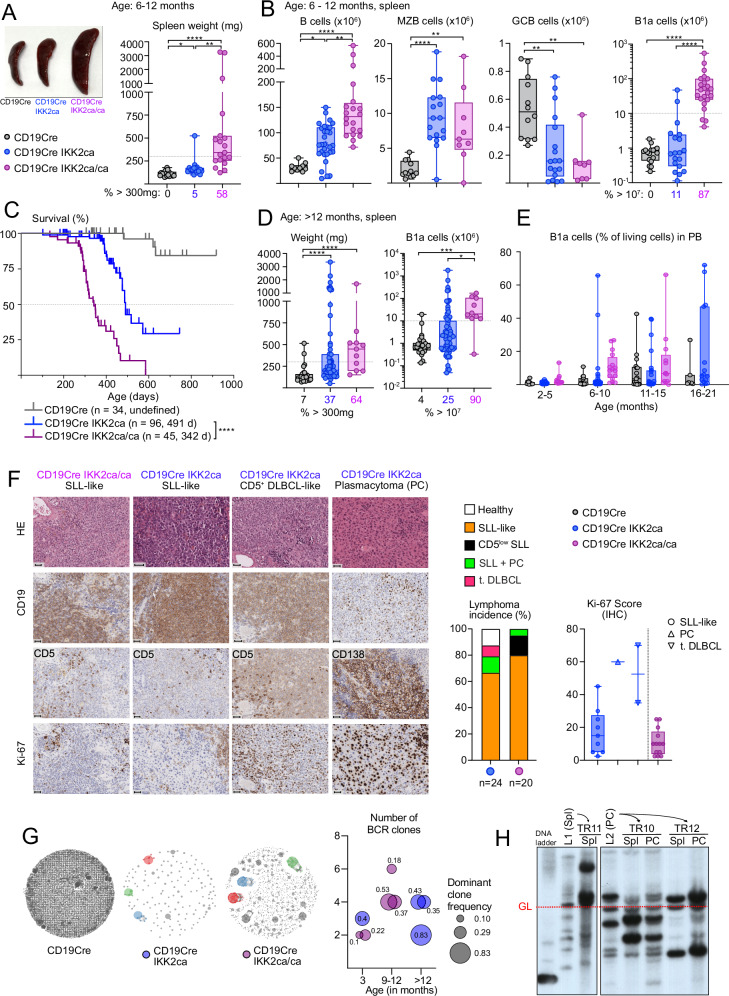
Dose-dependent induction of SLL/CLL-like lymphomagenesis by constitutive IKK2 signaling. **A**, **B** Ex vivo analysis of spleens from aged CD19Cre IKK2ca and CD19Cre IKK2ca/ca mice compared to CD19Cre control mice (6–12 months old). **A** Photograph of representative spleens from different genotypes and spleen weight. Percentages of mice with spleen weight above 300 mg are indicated below the graph for each genotype. **B** Absolute numbers of B cells, marginal zone B cells (MZB), germinal center B cells (GCB) and B1a cells in spleens of mice of the indicated genotypes. Percentages of mice with splenic B1a cell numbers above 10 million are indicated below the graph for each genotype. **C** Kaplan-Meier survival curve of CD19Cre IKK2ca compound mice, number of mice, their genotypes and median survival are indicated. **D** Spleen weight and absolute splenic B1a cell numbers of aged mice (>12 months). Percentage of mice with spleen weight above 300 mg or B1a cell numbers above 10 million are indicated below each graph for each genotype. **E** Percentage of B1a cells in peripheral blood (PB, % of living cells) of mice at different time points measured by flow cytometry. **F** Histological analysis of CD19cre IKK2ca lymphomas. Representative HE and immunohistochemistry (IHC) stainings of spleen sections for samples with small lymphocytic lymphoma-like (SLL) morphology, as well as transformed diffuse large B cell lymphoma-like (t.DLBCL) and plasmacytoma-like (PC) are shown for the indicated genotypes. The incidences of lymphoma and Ki-67 scores calculated by IHC are shown for the indicated genotypes. **G** BCR clonality analysis. Representative BCR clonality plots are shown for the indicated genotypes. IgM BCR clonality was assessed by RNA sequencing of B1a cells from mice of the indicated genotypes. Each dot represents a unique CDR3 sequence supported by ≥5 reads; dot size reflects read count. Expanded clones (>5% of functional reads) are shown as larger colored dots; non-clonal sequences appear as small gray dots. BCR clone counts and dominant clone frequency (size of circle) are depicted for each animal per genotype and age cohort. **H** Clonality assessment by Southern blot analysis of StuI-digested DNA isolated from primary lymphomas from CD19cre IKK2ca and CD19cre IKK2ca/ca and lymphomas expanded in adoptive transfer experiments. The employed JH probe detects IgH VDJ rearrangements. Arrows indicate the transfer of primary lymphomas (L) to transferred lymphomas (TR) expanding in individual recipient mice. GL germline, Spl spleen, PC peritoneal cavity. **p ≤ 0.01, ***p ≤ 0.001, ****p ≤ 0.0001, one-way ANOVA followed by Tukey’s multiple comparison test or Kruskal Wallis test followed by Dunn’s multiple comparison test. For full statistical analysis, see Table S2. B (CD19^+^), B2 (B220^hi^ CD19^+^), B1 (B220^low^ CD19^hi^), B1a (B220^low^ CD19^hi^ CD5^+^), B1b (B220^low^ CD19^hi^ CD5^-^), GCB (CD19^+^ CD95^hi^ CD38^low^) and MZB (CD19^+^ CD1d^hi^ CD21^+^CD23^low^).

To evaluate the oncogenic potential of graded constitutive IKK2-mediated canonical NF-κB activity in B cells, we monitored cohorts of mice for up to 20 months. At 6–12 months of age, CD19Cre IKK2ca/ca mice showed pronounced splenomegaly and B cell hyperplasia (Fig. 2A, B), which did not significantly impact numbers or distribution of CD4 and CD8 T cell subsets or different myeloid populations (Fig. S2A, B). MZB cells were not further expanded compared to CD19Cre IKK2ca mice. GCB cells were reduced in CD19Cre IKK2ca and even more in CD19Cre IKK2ca/ca mice (Fig. 2B), with a trend towards more plasma cells (Fig. S2C) in line with previous reports that constitutive NF-κB activation can interfere with germinal center reactions while favoring plasmacytic differentiation [9, 22]. The B1a cell expansion dramatically progressed in the immunological periphery of CD19Cre IKK2ca/ca mice (Figs. 2B and S2D), reaching 10–500 million in the spleens in the majority of the mice (Fig. 2B). Correspondingly, CD19Cre IKK2ca/ca mice displayed premature lethality, with a median survival of 342 days. CD19Cre IKK2ca mice lived on average 149 days longer (491 days, Fig. 2C) and at six to twelve months of age only rarely presented with splenomegaly and B1a cell expansion (Figs. 2B and S2D), both which occurred more frequently in older mice (Fig. 2D). Indolent lymphoproliferations of CD5^+^ B cells are characteristic for human CLL and small lymphocytic lymphoma (SLL), with the latter lacking the diagnostic CD5^+^ B cell accumulation in the peripheral blood [48]. Lymphoproliferations in mice expressing one or two IKK2ca transgene copies in B cells rather resembled SLL, as most of them did not present clearly enhanced CD5^+^ B1a proportions in the blood and maintained normal white blood cell counts and hemoglobin levels (Figs. 2E and S2E). However, CD19Cre IKK2ca/ca mice showed significantly reduced platelet counts which could indicate the onset of bone marrow failure (Fig. S2E). Histopathological examination confirmed the presence of lymphoproliferations with high similarity to human SLL/CLL in all analyzed CD19Cre IKK2ca/ca mice (*n* = 20, Fig. 2F). The normal architecture of the spleen was slightly disrupted by uniform, small-to-medium sized neoplastic B cells characterized by round nuclei, dense chromatin and CD5 expression of varying intensities (Fig. 2F). In addition, some mice showed plasma cell expansions.

The phenotype of the aged CD19Cre IKK2ca mice was more variable. Approximately 30–40% of heterozygous mice developed splenomegaly and B1a cell expansion, however with a longer latency (12–20 months) compared to IKK2ca/ca homozygous mice (Figs. 2D and S2F). Histopathology revealed a SLL/CLL phenotype in 68% of the animals (*n* = 23). In two mice (9%) a high-grade lymphoma (DLBCL-like) morphology was observed in the spleen and 14% of the animals developed plasmacytoma-like pathology (Fig. 2F). Ki-67 staining by immunohistochemistry and flow cytometry revealed increased proliferative activity within splenic B1a cells in both CD19Cre IKK2ca and CD19Cre IKK2ca/ca mice with a large variability within the groups (Figs. 2F, S2G, and S3). CD19Cre IKK2ca/ca mice exhibited high Ki-67 rates much earlier at 36–52 weeks than CD19Cre IKK2ca mice, consistent with their accelerated disease onset. Transformation into aggressive lymphomas, observed in two CD19Cre IKK2ca mice, was associated with a strong increase in Ki-67^+^ cells (Figs. 2F and S3A).

B cell receptor (BCR) RNA sequencing (Fig. 2G, Table S5C) and Southern blot (Fig. 2H) demonstrated that both CD19Cre IKK2ca/ca and CD19Cre IKK2ca mice developed oligoclonal lymphomas with BCR clone numbers ranging from two to six across individual animals. Notably, there was considerable variability in clonal size and dominance. While some mice exhibited relatively balanced clonal distributions, others displayed a single predominant clone. The degree of polyclonality did not correlate with severity of disease or survival (Fig. 2G), however, these conclusions are limited by the small sample size. We detected highly frequent stereotypic immunoglobulin heavy chain (IgH) rearrangements (Table S5D) amongst whose recurrent complementarity determining regions 3 (CDR3) were also sequences (CMRYGNYWYFDVW, CMRYGSSYWYFDVW) frequently detected in TCL1^tg ^CLL [49] and in a SV40 large T antigen-driven CLL mouse model [50]. These IgH CDR3s belong to the most frequently detected IgH sequences in peritoneal and splenic B1a cells with reactivities against phosphocholine of oxidized phospholipids [51].

Eµ-TCL1^tg^ mice (TCL1^tg^) [52], the most established preclinical model for human CLL, develop oligoclonal lymphomas that grow out as monoclonal lymphomas upon adoptive transfer into recipient mice [49, 53, 54]. The same holds true for IKK2ca/ca-expressing lymphomas, which could be propagated in immunocompetent and immunodeficient recipients, leading to the selection of individual clones (Figs. 2H and S2H). The lymphomas expanded more aggressively in subsequent transfers and infiltrated spleen, lymph nodes, liver and lung (data not shown). Taken together, constitutive B cell-specific NF-κB activation led to a lymphoproliferative disease with massive expansion of B1a cells in a dose-dependent manner. The observed phenotype resembles characteristics of human SLL/CLL and other preclinical CLL mouse models, especially TCL1^tg^ mice [23], which show a similar expansion of B1a cells in lymphoid tissues and a similar life span reduction (Fig. S2I).

### Constitutive IKK2 signals synergize dose-dependently with TCL1 expression in causing highly aggressive CLL-like disease

**Fig. 3:**
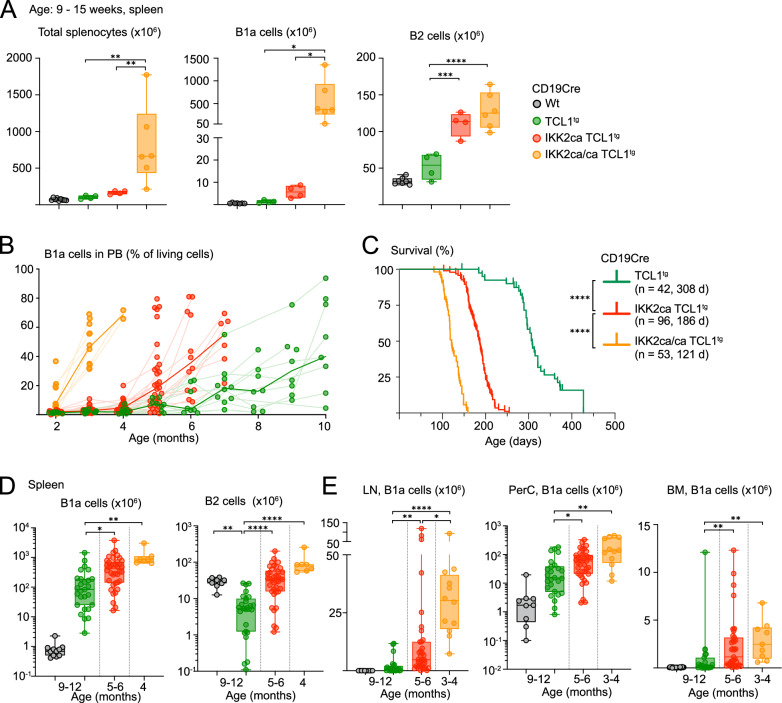
Dose-dependent synergy between IKK2ca and TCL1 expression in CLL-like lymphomagenesis. **A** Ex vivo analysis of young mice (9–15 weeks old) showing total splenocytes, absolute B1a and B2 cell numbers in spleen. **B** Scatter plot showing the percentage of B1a cells in peripheral blood (PB) over time, measured by flow cytometry analysis (% of living cells). The bold lines connect the median for each time point; the faint lines connect consecutive blood analyses from one mouse. **C** Kaplan-Meier survival curves, number of mice and median survival for each genotype are indicated. **D** Ex vivo analysis of spleens of burdened mice showing absolute numbers of B1a and B2 cells. **E** Ex vivo analysis of lymph nodes (LN), peritoneal cavity (PerC) and bone marrow (BM) from burdened mice showing absolute numbers of B1a cells measured by flow cytometry. **D**, **E** Age of burdened mice is indicated for each genotype. *p < 0.05, ** *p* ≤ 0.01, ***p ≤ 0.001, ****p ≤ 0.0001, one-way ANOVA followed by Tukey’s multiple comparison test or Kruskal Wallis test followed by Dunn’s multiple comparison test. For full statistical analysis, see Table S2. B (CD19^+^), B1a (CD19^hi^ B220^low^ CD5^+^), B2 (B220^hi^ CD19^+^).

Having identified that constitutive NF-κB activity in B cells triggers SLL/CLL-like disease in a dose-dependent fashion, we investigated whether IKK2ca expression would synergize with TCL1 overexpression, which was reported to also activate NF-κB [52, 55]. Below three months of age, CD19Cre IKK2ca/ca TCL1^tg^ mice already displayed a dramatic expansion of CD5^+^ IgM^+^ IgD^–^ B220^low^ B1a cells (Fig. 3A), which appeared in the peripheral blood at two months and continuously expanded (Fig. 3B) causing premature death with a median survival of 121 days (Fig. 3C). Between 3 and 4 months, when the mice became moribund with a pronounced splenomegaly, they exhibited massive infiltrations of malignant CD5^+^ B cells in spleen, lymph nodes, peritoneal cavity and bone marrow (Figs. 3D, E and S4A–C). CD19Cre IKK2ca TCL1^tg^ mice developed a similar phenotype 2–3 months later, although the malignant B1a cell expansion and infiltration did not reach the same levels. These parameters were further reduced in burdened CD19Cre TCL1^tg^ mice at around 9 months (median survival of 308 days). Burdened CD19Cre TCL1^tg^ mice had less B2 cells compared to CD19Cre controls, an effect which was compensated by IKK2ca expression and reversed by the expression of two copies (Figs. 3D and S4B, C). We observed trends for more CD4 T cells of all subsets, including Treg cells as well as more naive- and memory-like CD8 T cells in TCL1^tg^ mice with increased canonical NF-κB signaling (Fig. S4D), while myeloid cell populations did not seem to be altered in a consistent or significant manner within the TCL1^tg^ cohorts at the respective time of disease development (Fig. S4E).

**Fig. 4:**
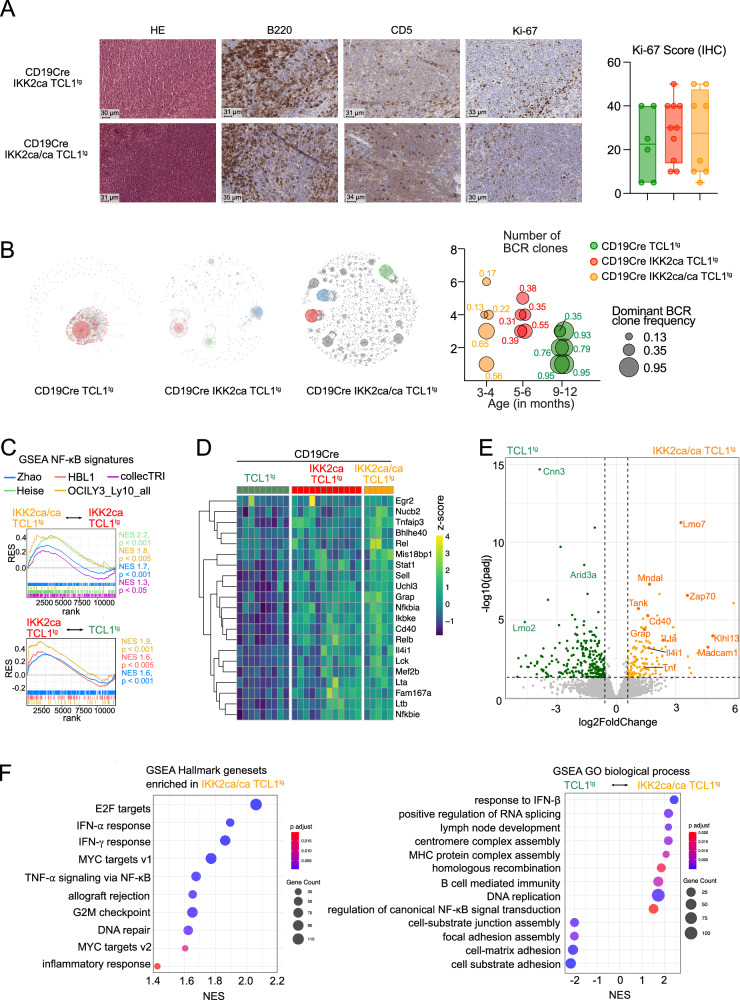
Impact of constitutive canonical NF-κB activation on histopathology, BCR clonality, and transcriptomes of TCL1^tg^ lymphomas. **A** Representative HE and immunohistochemistry (IHC) stainings of spleen sections and Ki-67 score calculated by IHC are shown for the indicated genotypes. **B** BCR clonality analysis. Representative BCR clonality plots are shown for the indicated genotypes. IgM BCR clonality was assessed by RNA sequencing of B1a cells from mice of the indicated genotypes. Each dot represents a unique CDR3 sequence supported by ≥5 reads; dot size reflects read count. Expanded clones (>5% of functional reads) are shown as larger colored dots; non-clonal sequences appear as small gray dots. BCR clone counts and dominant clone frequency (size of circle) are depicted for each animal per genotype and age cohort. **C–F** Bulk RNA sequencing of B1a cells from lymphomas from burdened mice. **C**) Gene set enrichment analysis (GSEA) of different published NFκB target gene signatures (listed in Table S3A) in (top) IKK2ca/ca TCL1^tg^ compared to IKK2ca TCL1^tg^ and (bottom) IKK2ca TCL1^tg^ lymphomas compared to TCL1^tg^ lymphomas. **D** Heatmap showing significantly upregulated NFκB target genes (log2FC > 0.58, adjusted *p* value <0.05) in IKK2ca/ca TCL1^tg^ mice compared to TCL1^tg^ mice. **E** Volcano plot showing differentially expressed genes in lymphomas of IKK2ca/ca TCL1^tg^ mice compared to TCL1^tg ^mice. **F** GSEA of hallmark gene sets and gene ontology (GO) terms “biological process” in IKK2ca/ca TCL1^tg^ lymphomas compared to TCL1^tg^ lymphomas. For GSEA statistics, see Table S3C, D, E; all differentially expressed genes are shown in Table S6. RES: running enrichment score, NES: normalized enrichment score, rank: rank in gene list (ranked by stat).

Histologic analyses revealed a disruption of the splenic architecture by small uniform B cells with increased proliferation activity in >80% of the heterozygous animals (*n* = 14) and in all homozygous mice (*n* = 10) (Fig. 4A). One CD19Cre IKK2ca TCL1^tg^ mouse displayed transformation in a highly proliferative high-grade lymphoma phenotype. Moreover, Ki-67 staining by immunohistochemistry revealed varying levels of proliferative activity within the CLL-like lymphomas, ranging from 5–50%, with a slight trend towards increased proportions of Ki-67^+^ cells when canonical NF-κB is activated (Figs. 4A and S4F).

BCR RNA sequencing (Fig. 4B, Table S5C) and IgH VDJ rearrangement sequencing (Table S5A) revealed oligoclonal lymphomas with a trend towards a higher number of clones in IKK2ca-expressing TCL1^tg^ lymphomas. As expected, nearly all clones contained unmutated (defined as >95% identity with the germline) and often stereotypic IgH VDJ sequences. They shared CDR3s with IKK2ca- and IKK2ca/ca-expressing, TCL1^tg^ and SV40LT-driven CLL cells and were highly prevalent in B1a cells [49–51, 56]. The stereotypic CDR3s are also found in BCRs recognizing phosphatidylcholine [57], oxidized lipids [51] and acrolein [56], indicating selection by modified autoantigens (Table S5D). For further characterization, we performed RNA sequencing on 5–12 FACS-purified lymphomas of the three genotypes (Fig. 4C–F). Overall, the lymphomas continued to express NF-κB target gene signatures corresponding to their IKK2ca copy number (Figs. 4C and S5A). A subset of core NF-κB target genes (*Nfkbia*, *Nfkbie*, *Tnfaip3*, *Cd40*, *Il4i1*, *Relb*, *Lta*, and *Ltb*) were differentially expressed in both premalignant B1a cells (Fig. 1F) and lymphomas (Fig. 4D), while other targets differed, likely related to the expression of TCL1, the transformation, and an altered microenvironment. We confirmed elevated expression of CD86 and CD40 on IKK2ca-expressing TCL1^tg^ compared to TCL1^tg^ B1a cells from spleen and lymph nodes (Fig. S5B).

In addition, to mimic T cell-mediated NF-κB activation within the lymph node microenvironment of human CLL, we co-cultured primary human CLL cells with follicular dendritic cells overexpressing CD40L and IL-21 (FDC CD40L IL-21). 20 patient-derived CLL samples were cultured on FDC CD40L IL-21, or for comparison, on EL08 stroma cells. Secretome analysis by mass spectrometry revealed a significantly increased secretion of IL4I1, LTA, soluble CD86 as well as PD-L2 under NF-κB-activating conditions (Fig. S5C)—factors that were also among the key upregulated genes in IKK2ca/ca expressing mice. Furthermore, we found the expression of *Zap70* and LIM domain only 7 protein (*Lmo7*), which are both highly expressed in IGHV unmutated CLL [58–60], to be upregulated in IKK2ca/ca TCL1^tg^ versus TCL1^tg^ lymphomas (Fig. 4E, Table S6A). Elevated *Lmo7* expression has been associated with enhanced migration and metastasis in several solid tumors [61]. Notably, correlations of CLL survival with RNA sequencing data from Knisbacher et al. using cBioPortal revealed an association of high *Lmo7* expression with reduced failure-free survival in CLL patients (Fig. S5D) [62–65]. Fitting with our results, proteogenomic studies [59] revealed high expression of LMO7 and CD40 specifically in CLL patients harboring BIRC3 mutations (Fig. S5E), one of the NF-κB-enhancing alterations found in CLL.

Gene Set Enrichment Analysis (GSEA) of hallmark gene sets and Gene Ontology Biological Processes of IKK2ca/ca TCL1^tg^ compared to TCL1^tg^ lymphomas revealed an upregulation of pathways associated with the cell cycle (“E2F targets”, “G2/M checkpoint”, “DNA replication”), enhanced inflammatory responses and immunomodulation (“IFN response”, “MHC protein complex assembly”), as well as potential DNA damage (“centromere complex assembly”, “DNA repair”, “homologous recombination”) (Figs. 4F and S5F). This underscores the fast-progressing phenotype of IKK2ca/ca TCL1^tg^ lymphomas and suggests that additional pathways are deregulated and contribute to their phenotype, including a downregulation of adhesion-related pathways. Consistent with this, gene signatures associated with adverse prognosis and proliferative drive in human CLL were significantly enriched in IKK2ca/ca TCL1^tg^ lymphomas (Fig. S5G). These include genes of the poor-prognosis cluster C2 [66], such as *Egr2*, *Tnf* and *Cd83*, genes upregulated in proliferating CLL cells in the lymph nodes [13], including NF-κB targets and E2F targets as well as Myc target genes that are upregulated in CLL cells with a proliferative drive (CLL-PD [67]). Importantly, these gene signatures were already enriched in peritoneal B1a cells of CD19Cre IKK2ca/ca mice compared to CD19Cre mice (Fig. S5H). Moreover, gene signatures up- or downregulated in murine and human Richter syndrome [68–71] were concordantly enriched or depleted in IKK2ca/ca TCL1^tg^ lymphomas, suggesting the rapid development of an aggressive lymphoma (Fig. S5I).

Comparative genome hybridization (CGH) detected trisomy 15 in most analyzed TCL1^tg^ and IKK2ca TCL1^tg^ samples (Fig. S5J), which is consistent with a gain of the c-Myc oncogene as previously reported [54]. In contrast, only one out of three IKK2ca/ca TCL1^tg^ lymphomas had a gain of chromosome 15, although they showed an upregulation of MYC targets in the GSEA (Fig. 4F), suggesting that the strong NF-κB activity obviates the need for genetic gain of Myc to some extent.

Taken together, our findings demonstrate that constitutive NF-κB signaling displays dramatic synergy with TCL1 overexpression in inducing a highly aggressive lymphoma with enhanced characteristics of unmutated CLL.

### IKK2 signals provide an immense cell-intrinsic competitive advantage to B1a cells, especially in the context of TCL1 expression

**Fig. 5:**
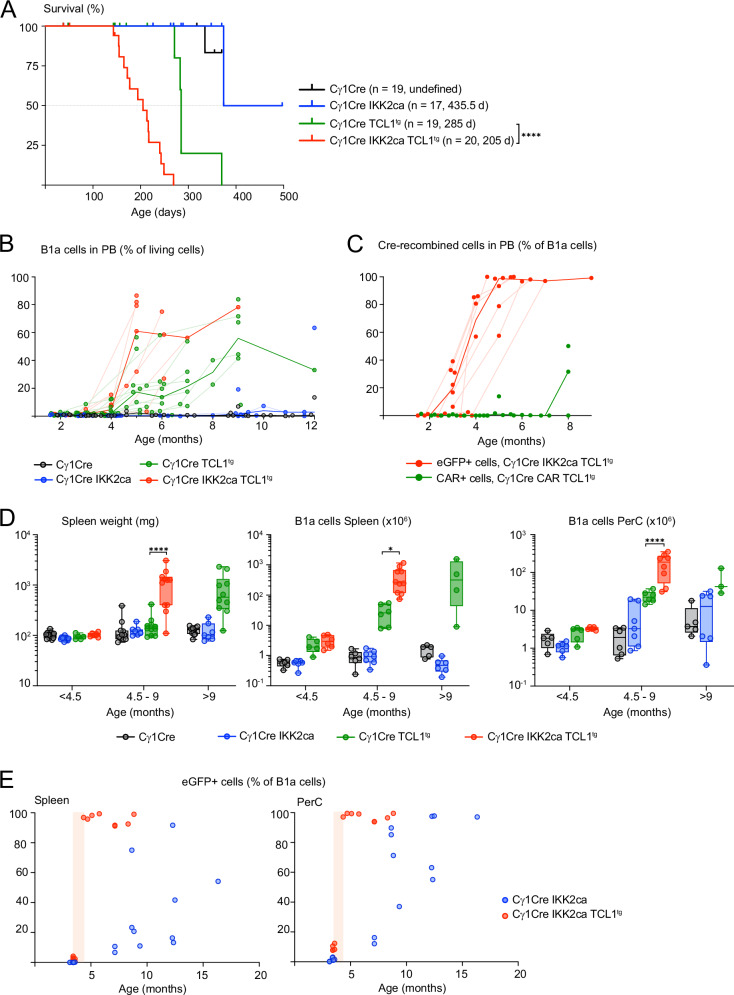
IKK2ca provides an immense cell-intrinsic competitive advantage to B1a cells, especially together with TCL1 expression. **A** Kaplan-Meier survival curves of Cγ1Cre IKK2ca TCL1^tg^ compound mice, genotypes, number of mice and median survival are indicated. **B** Scatter plot shows the percentage of B1a cells (% of living cells) in peripheral blood (PB) over time for the indicated genotypes. **C** Scatter plot shows the percentage of Cre-recombined cells (eGFP^+^ and CAR^+^) in peripheral blood as percentage of B1a cells measured by flow cytometry. **B**, **C** The bold lines connect the median of each time point. The faint lines connect consecutive blood analyses from one mouse. **D** Spleen weight and absolute numbers of splenic and peritoneal cavity (PerC) B1a cells in mice analyzed at different time points compared to age-matched controls. (Cγ1Cre IKK2ca: 4.5–16 months old, Cγ1Cre TCL1^tg^: 4.5–12 months old and Cγ1Cre IKK2ca TCL1^tg^: 4.5–9 months old). **E** Percentage of Cre-recombined B1a cells (eGFP^+^ cells as percentage of B1a cells) in spleen and peritoneal cavity of Cγ1Cre IKK2ca and Cγ1Cre IKK2ca TCL1^tg^ mice dependent on age. Shaded red area added to highlight the drastic increase in recombination. *p < 0.05, **p ≤ 0.01, ***p ≤ 0.001, ****p ≤ 0.0001, one-way ANOVA followed by Tukey’s multiple comparison test. Only selected comparisons are shown, for complete statistics see Table S2.

CLL can be subdivided into an IGHV unmutated (UM) and a mutated (M) subgroup, the latter is thought to have undergone a germinal center or germinal center-like reaction and is associated with a better prognosis [72]. TCL1^tg^ CLL represents a mouse model for unmutated CLL thus originating from pre-germinal center B cells. Given the strong oncogenic synergies between IKK2ca and TCL1, we aimed to assess whether expression of IKK2ca in activated B cells primed to enter germinal centers would generate a model for mutated CLL. For this purpose, we employed the Cγ1Cre knock-in transgene [25] and although activation of IKK2 in GCB cells leads to premature termination of the GCs in immunization experiments, IKK2ca-expressing GCB cells are produced [9, 22]. In line with these reports and with our observations in CD19Cre IKK2ca mice (Fig. 2B), we noted a reduction in spontaneous GCB cells in 8–16 months old Cγ1Cre IKK2ca mice, with significantly reduced but clearly detectable proportions of IKK2ca-expressing GCB cells (Fig. S6A). Remarkably, Cγ1Cre IKK2ca TCL1^tg^ mice had a significantly reduced lifespan compared to Cγ1Cre TCL1^tg ^controls (Fig. 5A), comparable to CD19Cre IKK2ca TCL1^tg^ mice (Fig. S6B). This correlated with an earlier expansion of CD5^+^ B cells in the peripheral blood of Cγ1Cre IKK2ca TCL1^tg^ mice, starting at four months of age (Fig. 5B). Cγ1Cre also mediates recombination in 1–2% of B1 cells [25], which comprise CD5^+^ B1a cells. Accordingly, we barely detected IKK2ca expression in the few CD5^+^ B cells present in the peripheral blood of two-month-old Cγ1Cre IKK2ca TCL1^tg ^mice. However, at 3 months of age, just prior to the expansion of CD5^+^ TCL1^tg^ B cells, up to 40% of them expressed IKK2ca, which gradually increased to 100% by 5 months (Fig. 5C). This could reflect preferential expansion of minimal numbers of IKK2ca-expressing CD5^+^ TCL1^tg^ B cells but also on-going Cre-mediated recombination in the expanding B1a cell population. To better differentiate between these two possibilities, we generated Cγ1Cre CAR TCL1^tg^ mice expressing the coxsackie adenovirus receptor (CAR) from a Rosa26 knock-in transgene upon Cre-mediated recombination of the same loxP-flanked STOP cassette as used in the conditional IKK2ca allele [27]. Here, the expanding CD5^+^ TCL1^tg^ B cells lacked expression of the Cre-inducible CAR for up to eight months (Fig. 5C), indicating that the increase in IKK2ca-expressing TCL1^tg^ CD5^+^ B cells is most likely due to their preferential expansion. Analysis of mice at different timepoints revealed a two to three months earlier onset of splenomegaly and CD5^+^ B cell expansion in spleen and peritoneal cavity of Cγ1Cre IKK2ca TCL1^tg^ compared to Cγ1Cre TCL1^tg^ mice (Fig. 5D). Similar to the situation in the peripheral blood, IKK2ca-expressing CD5^+^ TCL1^tg^ B cells expanded from barely detectable proportions in spleen and peritoneal cavity to over 90% of the B1a cell population within one month (Fig. 5E), coinciding with their massive accumulation in these organs (Fig. 5D). IKK2ca expression equips CD5^+^ B cells with a cell-intrinsic competitive advantage independent of TCL1, as also in spleen and peritoneal cavity of Cγ1Cre IKK2ca mice the proportion of IKK2ca-expressing CD5^+^ B cells increases from background levels to over 90% over a timeframe of 10 months (Fig. 5E). In the peritoneal cavity, this corresponds to an expansion of mostly CD5^+^ B cells (Fig. 5D). As TCL1 overexpression can in principle induce GCB cell-derived lymphomas [73], we employed AIDCre [26] mice to exclude the possibility that particularities of the Cγ1Cre-mediated recombination prevented oncogenic collaboration between IKK2ca and TCL1 in GCB cells and GCB-derived cells. However, AIDCre IKK2ca TCL1^tg^ mice essentially phenocopied the phenotype of Cγ1Cre IKK2ca TCL1^tg^ mice with respect to survival (Fig. S6B, C) and CD5^+^ B cell expansion in the peripheral blood, spleen, and peritoneal cavity (Fig. S6D, E). Also, here, the fraction of IKK2ca-expressing TCL1^tg^ CD5^+^ B cells increased dramatically over a short period of time (Fig.S6D). Evaluation of AIDCre-mediated recombination using the very sensitive Cre reporter LSL-tdTomato [28] revealed low-level recombination in B1a, B1b, and B2 cells, in addition to nearly complete recombination in GCB cells (Fig. S6F and data not shown). While the proportion of IKK2ca-expressing GCB cells was low at 2–21% (data not shown), the proportion of IKK2ca-expressing B2 and B1b cells was increased compared to the corresponding proportions of tdTomato-expressing cells in AIDCre tdTomato mice and this effect was strongly enhanced in TCL1-expressing cells (Fig. S6F). This shows that the synergistic effect of constitutive IKK2 activation and TCL1 expression is not limited to B1a cells. Nevertheless, the strongest cell-intrinsic competitive advantage afforded by IKK2ca expression was again observed in B1a cells, especially in combination with TCL1 expression (Fig. S6E).

In summary, we show that IKK2ca-induced signaling equips CD5^+^ B cells with an enormous cell-intrinsic competitive advantage, such that a minute number of IKK2ca-expressing TCL1^tg^ CD5^+^ B cells quickly outcompete all other TCL1^tg^ B cells and cause deadly CLL-like disease with the same kinetics as in mice where virtually all B cells co-express TCL1 and IKK2ca.

### High constitutive IKK2 signaling replaces obligate microenvironmental maintenance cues in CLL-like disease

CLL cells are highly microenvironment-dependent and require the presence and actions of many supporting cell types with whom they engage in complex bi-directional cross-talk [74]. Many of those are implicated in inducing NF-κB activation in CLL [13, 15]. This raises the question of whether constitutive cell-intrinsic NF-κB activation could reduce or obviate CLL’s microenvironment dependence.

**Fig. 6:**
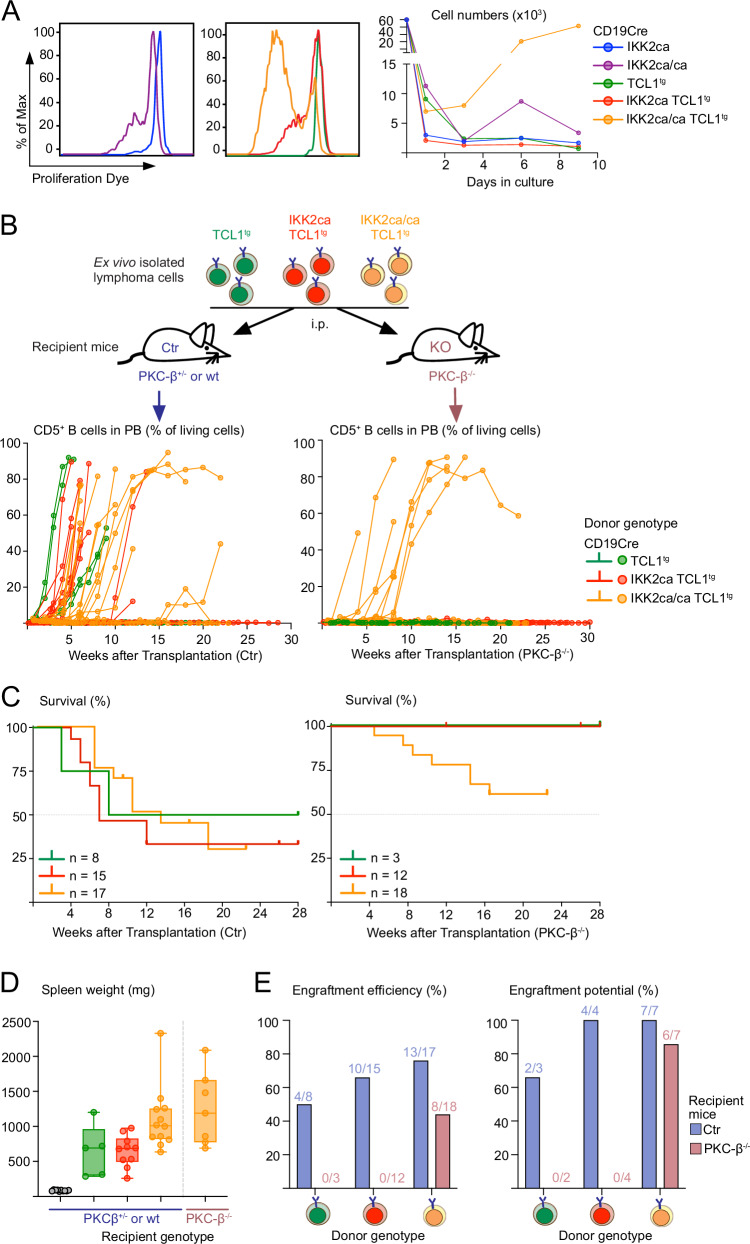
Strong canonical NF-κB activation overcomes microenvironment dependencies of TCL1^tg^ lymphomas. **A** In vitro proliferation of FACS-purified peritoneal B1a cells of the indicated genotypes when cultured in vitro for 9 days without stimulation. Proliferation was assessed by flow cytometry using the eFluor450 Proliferation Dye. Representative flow cytometry histograms and scatter plot of absolute numbers of living B1a cells in culture are shown (representative data from at least four independent experiments). **B** Experimental scheme: MACS-purified splenic B cells form burdened CD19Cre TCL1^tg^, CD19Cre IKK2ca TCL1^tg^ and CD19Cre IKK2ca/ca TCL1^tg^ mice were transplanted into PKC-β proficient (PKC-β^+/-^ and wt = control, Ctr) and PKC-β knockout (PKC-β^−/−^) recipient mice by intraperitoneal injection (i.p.) of 20 million cells. Mice were monitored for 6 months, burdened mice were sacrificed and further analyzed. Scatter plots show the percentage of B1a cells in peripheral blood (PB) after transplantation into PKC-β proficient (left) or deficient (right) mice measured by flow cytometry. The lines connect measurements at different time points for each mouse. **C** Kaplan-Meier-survival curves post-transplant, number of mice and median survival are indicated. **D** Spleen weight of burdened recipient mice. Donor and recipient genotypes are annotated. **E** Engraftment efficiency (Percentage of transplantations that led to successful engraftment defined by expansion of GFP^+^ donor B cells in peripheral blood) and engraftment potential (percentage of lymphomas that could engraft) are depicted. *p < 0.05, **p ≤ 0.01, ***p ≤ 0.001, ****p ≤ 0.0001, Kruskal Wallis test followed by Dunn’s multiple comparison test.

In a first step, we cultured premalignant B1a cells expressing one or two transgene copies of IKK2ca with or without TCL1. IKK2ca-expressing TCL1^tg^ as well as IKK2ca/ca-expressing B1a cells displayed low-level spontaneous cell division in vitro, but their numbers quickly decreased (Fig. 6A). In contrast, a large proportion of IKK2ca/ca-expressing TCL1^tg^ B1a cells proliferated vigorously, and after an initial drop, they continuously expanded for up to ten days.

Upon adoptive transfer, TCL1^tg^ lymphoma cells engraft and expand in immunocompetent recipient mice [53]. We previously showed that the long-term expansion, but not the initial homing of TCL1^tg^ CLL cells, critically depends on the presence of protein kinase C-β (PKC-β) in non-lymphoma cells, as TCL1^tg^ CLL cells do not stably engraft in PKC-β^−/−^ mice [75, 76]. Non-hematopoietic cells and especially mesenchymal stromal cells, which are of critical importance in CLL maintenance [74], play a dominant role in this process via CLL-induced upregulation of the PKC-βII splice isoform. PKC-β, in turn, mediates extensive transcriptional and metabolic reprogramming of the stroma, thereby ensuring robust CLL support via various mechanisms [75, 76].

To evaluate the impact of IKK2ca-mediated NF-κB activation on the dependence of TCL1^tg^ CLL cells on this powerful support system, we transplanted TCL1-, IKK2ca TCL1- and IKK2ca/ca TCL1-expressing CLL cells in parallel into control (wild-type or PKC-β^+/-^ littermates) and PKC-β^−/−^ recipient mice (Fig. 6B). The expansion of the CD5^+^ lymphoma cells in the peripheral blood of the recipient control mice had variable kinetics and caused reduced survival without clear differences between the genotypes (Fig. 6B, C), although IKK2ca/ca TCL1^tg^ CLL cells induced more pronounced splenomegaly (Fig. 6D), consistent with the autochthonous model. Overall, in control recipients, the engraftment efficiency (% of transplanted mice in which lymphoma cells expanded) increased slightly with increasing IKK2ca dose (Figs. 6E and S7). With the exception of TCL1^tg^ lymphomas, where one lymphoma failed to engraft altogether, all investigated lymphomas engrafted in at least one recipient (100% engraftment potential). In striking contrast, while none of the TCL1^tg^ and IKK2ca TCL1^tg^ lymphomas engrafted in PKC-β^−/−^ recipients (Fig. 6B–E), IKK2ca/ca TCL1^tg^ CLL cells expanded in PKC-β^−/−^ mice with similar kinetics as in controls (Fig. 6B). Although the engraftment efficiency of IKK2ca/ca TCL1^tg^ CLL cells was reduced by 50% in PKC-β^−/−^ compared to control recipient mice, but six out of seven transplanted lymphomas engrafted in at least one PKC-β^−/−^ recipient (Figs. 6E and S7), demonstrating that strong constitutive NF-κB activation in TCL1^tg^ CLL cells suffices to overcome critical requirements for environmental support.

In conclusion, we demonstrate that strong constitutive canonical NF-κB signaling suffices to overcome microenvironmental dependencies in a mouse model for CLL, which probably represents a key mechanism explaining the extremely aggressive disease course of CD19Cre IKK2ca/ca TCL1^tg^ mice.

## Discussion

NF-κB signaling plays a major role in CLL pathogenesis, mainly through complex external activation from various cellular sources of its microenvironment [13, 15, 75]. NF-κB activation is also associated with disease progression and drug resistance [16]. Our results, modeling strong canonical NF-κB signaling in all B cells, prove that it can be a direct oncogenic event. This is particularly true for B1a cells, as constitutive NF-κB signals trigger a disease highly similar to the TCL1^tg^ preclinical CLL mouse model. Unlike B2 cells, which expanded linearly with increased IKK2ca dosage, in B1a cells, we observed a threshold effect where high canonical NF-κB activity was required for their expansion. The preferential transformation of B1a cells, which were proposed as a candidate for the cell-of-origin in human CLL [77, 78], could be linked to NF-κB-mediated enhancement of their self-renewing properties [31, 79, 80]. Moreover, the observed pattern of recurrent stereotypic BCRs in lymphoma clones indicates that BCR selection, which is central to lymphomagenic B1a cell expansion in mice [31, 80] and men [81, 82], contributes to the disease. Fitting with a concept that IKK2 signals enhance but cannot replace (auto-)antigenic signals during lymphomagenesis, BCR-induced proliferation is massively amplified by constitutive canonical NF-κB activation [8].

Constitutive IKK2 signals dose-dependently accelerate the CLL-like disease in TCL1^tg^ animals, increasing gene expression typical for IGHV unmutated CLL and Richter transformation. This can be attributed to our finding that constitutive canonical NF-κB activation equips B1a cells with a cell-intrinsic competitive advantage, which is dramatically amplified in conjunction with TCL1 overexpression.

Cell-intrinsic genetic alterations affecting the NF-κB pathway (including *NFKBIE*, *MYD88*, *NFKBIZ*, *BIRC3*, and *NOTCH1*) occur in about 10% of CLL patients [83, 84]. In addition, whole exome sequencing of 984 CLL patients identified low-frequency (<1%) mutations in *NFKBIB*, *RELA*, *IKBKB*, and *NFKB1* [62], and *NFKB1* mutations correlate with reduced failure-free and overall survival. *NFKBIE* mutations are the most frequent (occurring in around 7% of patients) NF-κB-related alteration in CLL and correlate with poor prognosis [85]. Subsequent murine studies showed that IκBε deficiency led to B1 cell expansions in various secondary lymphoid organs at 18 months of age [86], consistent with enhanced NF-κB activation in stimulated IκBε-deficient B cells [86, 87]. While *Nfkbie* knockout did not trigger B cell transformation [86, 88], it accelerated leukemogenesis in the TCL1^tg^ mouse model [88]. Furthermore, human CLL cells harboring CRISPR/Cas9-introduced *NFKBIE* mutations were enriched over time in vitro upon stimulation with CD40L and CpG (but not αIgM) [89]. We detected IKK2ca-mediated upregulation of *Nfkbia* and *Nfkbie* transcripts, reflecting part of the strict negative feedback control of the NF-κB pathways. The strong constant canonical NF-κB signals induce the instant degradation of re-expressed IκBα protein, thereby overcoming feedback inhibition through *Nfkbia*. However, IκBε protein levels remain high and can therefore dampen IKK2-driven NF-κB activation, which might explain the prevalence of *NFKBIE* mutations in CLL.

The B cell maintenance cytokine BAFF was reported to activate alternative and canonical NF-κB in CLL [90]. The fact that CLL patients have lower BAFF serum levels [91] could point to increased consumption by CLL cells. In line with this notion, BAFF overexpression accelerated CLL-like disease in TCL1^tg^ mice [92] and BAFF is required for the dissemination of TCL1^tg^ CD5^+^ B cells beyond the peritoneal cavity [93]. CLL-like cells, especially outside the peritoneal cavity, might therefore depend on BAFF for expansion and/or protection against apoptosis [94], and constitutive canonical NF-κB activation could replace these functions, as it replaces the requirement for BAFF-R signals in B2 cells [8].

Among the prototypical NF-κB-induced genes that we found to be upregulated, *Tnf* is of particular interest as TNF induces proliferation of CLL cells [95, 96], elevated serum levels in CLL patients indicate a more aggressive disease and correlate with poor survival [45, 97] and TNFR inhibition showed clinical efficacy in patients [98]. Moreover, LTA and IL4I1 were strongly upregulated in both our mouse model and the secretome of primary CLL cells under NF-κB-activating conditions. This supports observations in Hodgkin lymphoma, where LTA drives autocrine and paracrine activation of NF-κB and JAK2/STAT6 signaling and induces the expression of CD40 and PD-L2 [46], which were similarly upregulated in IKK2ca/ca expressing B1a cells. IL4I1 has been shown to promote CLL progression in the TCL1^tg^ mouse model by enhancing CD8 T cell exhaustion and supporting immunosuppressive myeloid-derived suppressor cells and Treg cells [44]. Consistent with this, we saw a trend towards upregulation of Treg cells in the spleens of IKK2ca and IKK2ca/ca TCL1^tg^ mice. IL4I1 production by non-lymphoma cells, for example splenic monocytes, was shown to promote murine CLL cell expansion [44]. Our data reveal that intrinsic NF-κB activation strongly enhances IL4I1 production by CLL cells themselves, which could render them less reliant on their microenvironment. In addition, IKK2ca/ca TCL1^tg^ lymphomas showed transcriptional changes that mirror known features of human CLL with proliferative drive and adverse prognosis. Among those, we saw a strong upregulation of LMO7 and ZAP70, both associated with IGHV-UM CLL. LMO7 expression, which is also elevated in BIRC3 mutant CLL, is associated with poor clinical outcomes. The underlying mechanisms remain unclear, however, it has been shown that LMO7 promotes tumor cell proliferation and migration in several solid malignancies [99, 100]. ZAP70, on the other hand, mediates CLL cell survival, protein synthesis, and tonic BCR signals via AKT [101–103].

Given that microenvironmental signals are the main inducers of NF-κB activation in CLL [15] but also engage additional pathways, the question arises whether intrinsic NF-κB signaling can reduce CLL’s dependency on signals from its microenvironment. The development of CLL in TCL1^tg^ mice does not strictly depend on CD40 signals or even the presence of T cells [80], but the maintenance of TCL1^tg^ CLL cells absolutely depends on PKC-β-mediated signals from their microenvironment. In PKC-β-deficient recipient mice, PKC-β-derived peptides presented by the transferred CLL could, in principle, be recognized as foreign antigens by host T cells. However, in our model, the transferred IKK2ca and IKK2ca/ca TCL1^tg^ CLL cells express the highly antigenic eGFP (and the potentially antigenic human TCL1), which should minimize differences in anti-CLL T cell responses between PKC-β-deficient and control recipients. Nevertheless, we cannot exclude that anti-GFP T cell responses could mask an in principle faster and more aggressive engraftment of IKK2ca-expressing TCL1^tg^ cells compared to TCL1^tg^ cells lacking eGFP in control recipients. Six out of seven tested primary IKK2ca/ca TCL1^tg^ lymphomas engrafted in PKC-β-deficient mice, which we take as proof that strong constitutive canonical NF-κB signals can effectively replace critical microenvironmental support. Thus, enhanced NF-κB activity might act as a resistance mechanism in CLL refractory to microenvironment-directed therapies. Indeed, microenvironmental-induced NF-κB activation was implicated in the resistance of CLL cells to venetoclax and ibrutinib [104]. Moreover, enhanced NF-κB activity has been associated with Richter syndrome, the transformation of CLL into an aggressive lymphoma [68, 105]. Consistent with this observation, we saw an enrichment of Richter syndrome gene signatures in IKK2ca/ca TCL1^tg^ mice highlighting the role of strong NF-κB signaling in driving aggressive disease.

In conclusion, we present a genetic mouse model in which cell-intrinsic constitutive NF-κB activation alone triggers B cell lymphomagenesis with high resemblance to human SLL/CLL. In conjunction with TCL1 overexpression, our model mimics key aspects of aggressive human CLL and other lymphomas, highlights an oncogenic mechanism towards microenvironmental independence and can serve as a valuable preclinical model in these areas.

## Supplementary information

Supplemental Information

Supplementary figures S1–S7

Supplemental Table 1 Resources

Supplemental Table 2 Statistics

Supplemental Table 3 GSEA

Supplemental Table 4 DEG young mice

Supplemental Table 5 IgH VDJ rearrangements

Supplemental Table 6 DEG lymphomas

## Data Availability

RNA sequencing data were deposited in the NCBI Gene Expression Omnibus (GEO) database; the GSE accession numbers are GSE289013 and GSE289016. The mass spectrometry proteomics data have been deposited in the ProteomeXchange Consortium via the PRIDE partner repository with the dataset identifier PXD069904.
