# Correction: Strong constitutive NF-κB signaling in B cells drives SLL/CLL-like lymphomagenesis and overcomes microenvironmental dependencies

**DOI:** 10.1038/s41375-026-02863-z

**Published:** 2026-02-11

**Authors:** Valeria Soberón, Lena Osswald, Andrew Moore, Dominika Sosnowska, Gene Swinerd, Jingyu Chen, Seren Baygün, Carina Diehl, Gönül Seyhan, Laura Kraus, Vanessa Gölling, Ricarda Trapp, Thomas J. O’Neill, Sabrina Bortoluzzi, Daniel Kovacs, Tim Ammon, Pankaj Singroul, Yuliia Hubarzhevska, Rupert Öllinger, Sebastian Mueller, Olga Baranov, Piero Giansanti, Felix Gillhuber, Sonja Grath, Oliver Weigert, Andreas Rosenwald, Yoshiteru Sasaki, Klaus Rajewsky, Katja Steiger, Florian Bassermann, Roland Rad, Daniel Krappmann, Ingo Ringshausen, Marc Schmidt-Supprian

**Affiliations:** 1 Institute for Experimental Hematology, School of Medicine and Health, Technical University of Munich, Munich, Germany; 2 Center for Translational Cancer Research (TranslaTUM), School of Medicine and Health, Technical University of Munich, Munich, Germany; 3 German Cancer Consortium (DKTK), Heidelberg, Germany; 4 Max-Planck Institute of Biochemistry, Planegg, Germany; 5 Department of Medicine III, School of Medicine and Health, Technical University of Munich, Munich, Germany; 6 Department of Haematology, University of Cambridge, Cambridge, UK; 7 Cancer Institute, University College London, London, UK; 8 Department of Biochemistry and Molecular Biology, West China School of Basic Medical Sciences & Forensic Medicine, Sichuan University, Chengdu, PR China; 9 Research Unit Signaling and Translation, Molecular Targets and Therapeutics Center, Helmholtz Zentrum München-German Research Center for Environmental Health, Neuherberg, Germany; 10 Institute of Molecular Oncology and Functional Genomics, School of Medicine and Health, Technical University of Munich, Munich, Germany; 11 Bavarian Center for Biomolecular Mass Spectrometry at Klinikum rechts der Isar, School of Medicine and Health, Technical University of Munich, Munich, Germany; 12 Division of Evolutionary Biology, Faculty of Biology, Ludwig-Maximilians-Universität (LMU) München, Planegg-Martinsried, Germany; 13 Laboratory for Experimental Leukemia and Lymphoma Research (ELLF), Faculty of Medicine, Department of Medicine III, Ludwig-Maximilians-University, Munich, Germany; 14 Institute of Pathology, University of Würzburg, Würzburg, Germany; 15 Program in Cellular and Molecular Medicine, Children’s Hospital, and Immune Disease Institute, Harvard Medical School, Boston, MA USA; 16 Division of Immunology, Faculty of Medicine, Tohoku Medical and Pharmaceutical University, Sendai, Japan; 17 Immune Regulation and Cancer, Max-Delbrück-Center for Molecular Medicine in the Helmholtz Association (MDC), Berlin, Germany; 18 Institute of Pathology, School of Medicine and Health, Technical University of Munich, Munich, Germany; 19 Bavarian Cancer Research Center (BZKF), Munich, Germany

**Keywords:** Chronic lymphocytic leukaemia, B-cell lymphoma, Preclinical research, Lymphoproliferative disorders, B-1 cells

Correction to: *Leukemia* 10.1038/s41375-025-02844-8, published online 16 January 2026

After initial online publication, it was identified that Figures 2 and 4 did not reflect the correct final versions. These figures have now been replaced, and the article has been updated. The original article has been corrected.

