# Supplementary material for: Strong constitutive NF-κB signaling in B cells drives SLL/CLL-like lymphomagenesis and overcomes microenvironmental dependencies
Source: Leukemia. 2026 Jan 16;40(3):522–39. doi: 10.1038/s41375-025-02844-8 (PMC12960208; doi:10.1038/s41375-025-02844-8)
Supplement: Supplementary file 1 — Supplemental Information [file 41375_2025_2844_MOESM1_ESM.pdf]

Supplementary Information

Supplementary Figures

Supplementary Figure S1
Effects of B cell specific constitutive IKK2 signaling in young mice

Supplementary Figure S2
Constitutive IKK2 activity induces lymphomagenesis in a dose-dependent manner

Supplementary Figure S3
Ki-67 staining of IKK2ca-driven lymphomas reveals variable proliferative activity

Supplementary Figure S4
B cell-intrinsic and -extrinsic consequences of synergistic effects of IKK2ca and TCL1 co-expression

Supplementary Figure S5
Transcriptomic and chromosomal analysis of IKK2ca/ca- and TCL1-expressing lymphomas

Supplementary Figure S6
Competitive advantage of B1a cells expressing IKK2ca, alone and in combination with TCL1

Supplementary Figure S7
IKK2ca/ca-expressing TCL1tg CLL cells engraft in PKCβ-deficient mice

Supplementary Tables

Supplementary Table S1
Resources (primers and antibodies)

Supplementary Table S2
Statistical analyses of all Figures

Supplementary Table S3
Gene signatures used for GSEA and GSEA results

Supplementary Table S4
Differentially expressed genes in spleen and peritoneal cavity of young mice

Supplementary Table S5
IgH VDJ rearrangement analyses by PCR-cloning-sequencing and BCR RNA sequencing

Supplementary Table S6
Differentially expressed genes in lymphomas of aged mice
