# Supplementary material for: Strong constitutive NF-κB signaling in B cells drives SLL/CLL-like lymphomagenesis and overcomes microenvironmental dependencies
Source: Leukemia. 2026 Jan 16;40(3):522–39. doi: 10.1038/s41375-025-02844-8 (PMC12960208; doi:10.1038/s41375-025-02844-8)
Supplement: Supplementary file 2 — Supplementary figures S1–S7 [file 41375_2025_2844_MOESM2_ESM.pdf]

Supplemental Figure S1

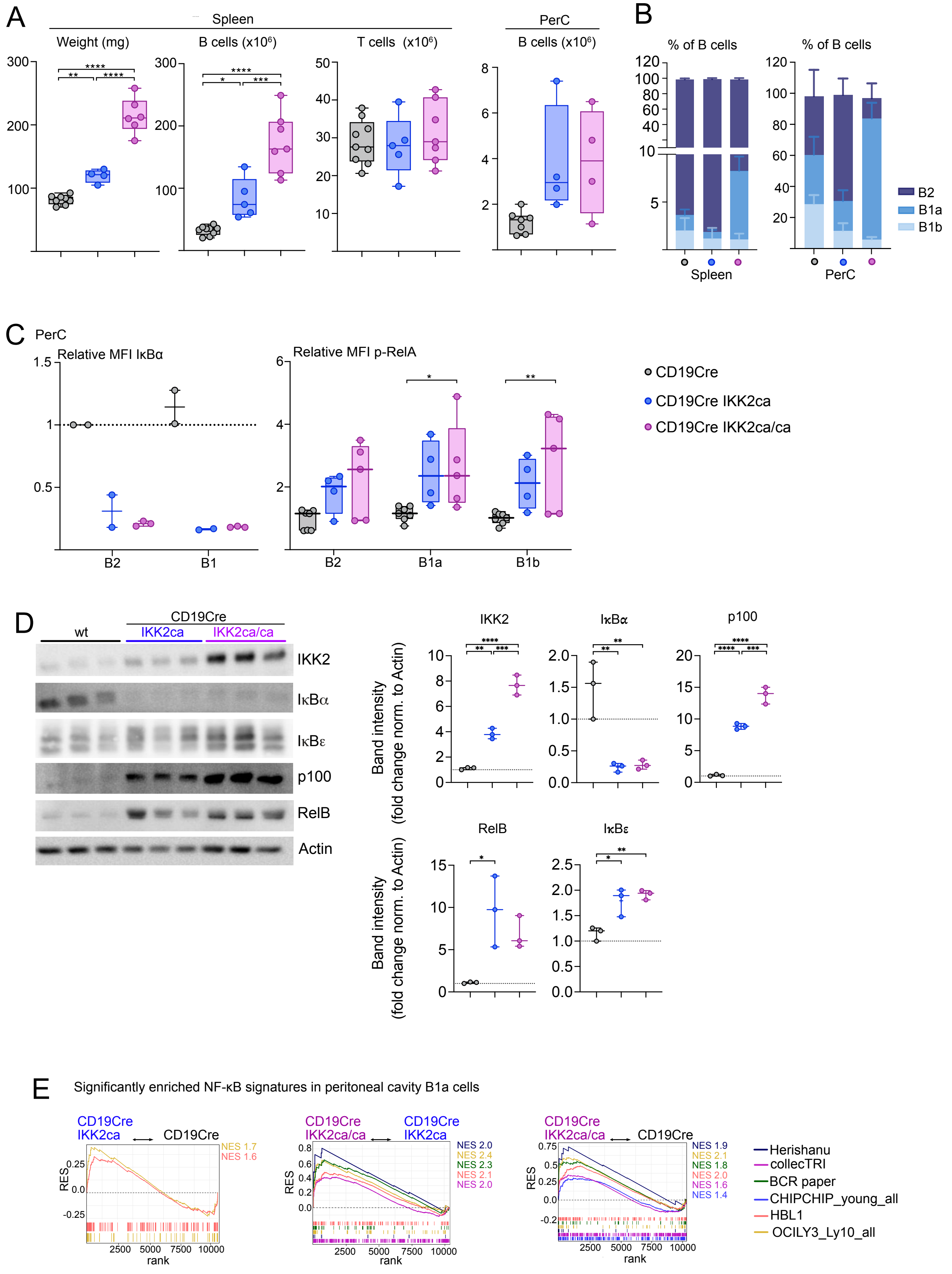

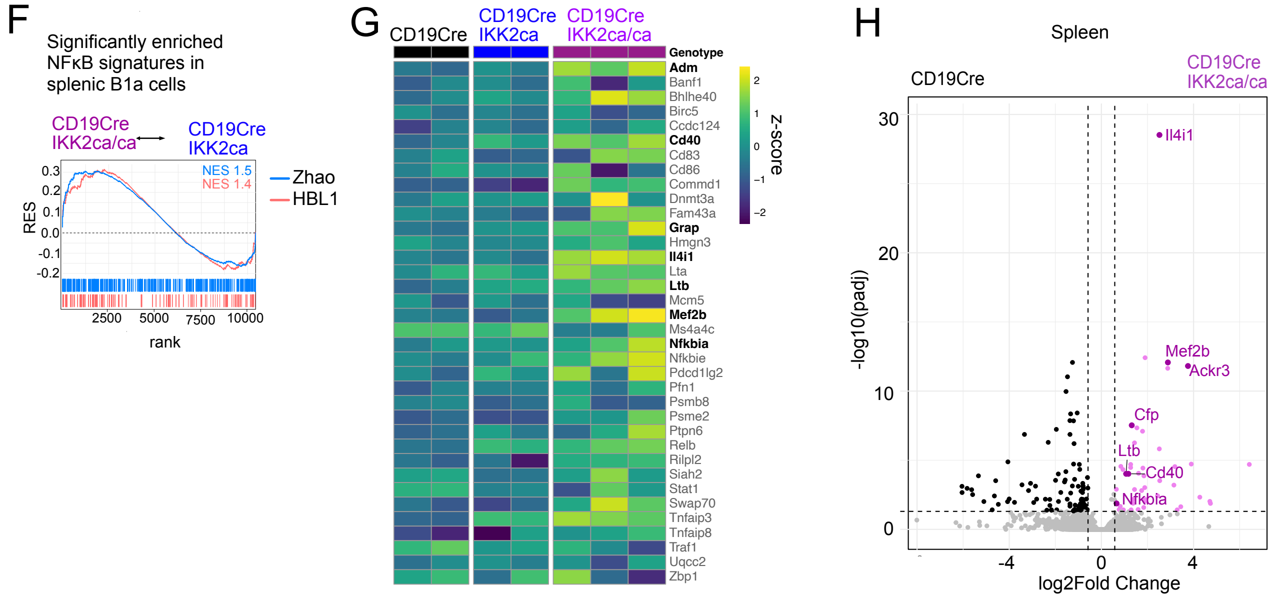

Suppl. Figure S1: Effects of B cell specific constitutive IKK2 signaling in young mice

A-B) *Ex vivo* flow cytometry analysis of spleen and peritoneal cavity (PerC) of 3 months old CD19Cre IKK2ca and CD19Cre IKK2ca/ca mice compared to CD19Cre control mice.

A) Spleen weight, absolute splenic B and T cell numbers, and absolute peritoneal B cell numbers.

B) Percentage of B2, B1a and B1b populations in spleen and peritoneal cavity. Mean values +/- SD are shown.

C) Intracellular flow cytometry analysis of IκBα degradation and phosphorylated RelA (p-RelA) levels in unstimulated freshly isolated peritoneal B cells from young mice (3-4.5 months old). Median fluorescent intensity (MFI) was normalized to CD19Cre control samples (IκBα) or T cells for each sample (p-RelA) (3 independent experiments).

D) Western blot analysis of MACS-purified splenic B cells of aged mice (wildtype: 6 months, CD19Cre IKK2ca and CD19Cre IKK2ca/ca: 9 months). Quantification of relative band intensities normalized to β-actin loading controls and to wild-type controls.

E) Bulk RNA sequencing analysis of FACS-purified peritoneal B1a cells from young mice. Gene set enrichment analysis (GSEA) of published NFκB target gene signatures (additional to Fig 1).

F-H) Bulk RNA sequencing analysis of FACS-purified splenic B1a cells from young mice.

F) GSEA plot showing significantly enriched NFκB signatures in CD19Cre IKK2ca/ca mice compared to CD19Cre IKK2ca mice.

G) Heatmap showing NFκB target genes that are significantly upregulated in peritoneal cavity. Genes highlighted in bold are also significantly upregulated in splenic B1a cells in IKK2ca/ca mice when compared to CD19Cre control mice (log2FC >0.58, adj p value <0.05).

H) Volcano plot showing differentially expressed genes in CD19Cre IKK2ca/ca mice compared to CD19Cre control mice.

* $p \leq 0.05$, ** $p \leq 0.01$, *** $p \leq 0.001$, **** $p \leq 0.0001$, one-way ANOVA followed by Tukey's multiple comparison test. For full statistical analysis see Table S2.

Gene signatures used for GSEA are shown in Table S3A, GSEA statistics are shown in Table S3B. Differentially expressed genes are shown in Table S4.

RES = running enrichment score, NES = normalized enrichment score, rank = rank in gene list (ranked by stat)

Supplemental Figure S2

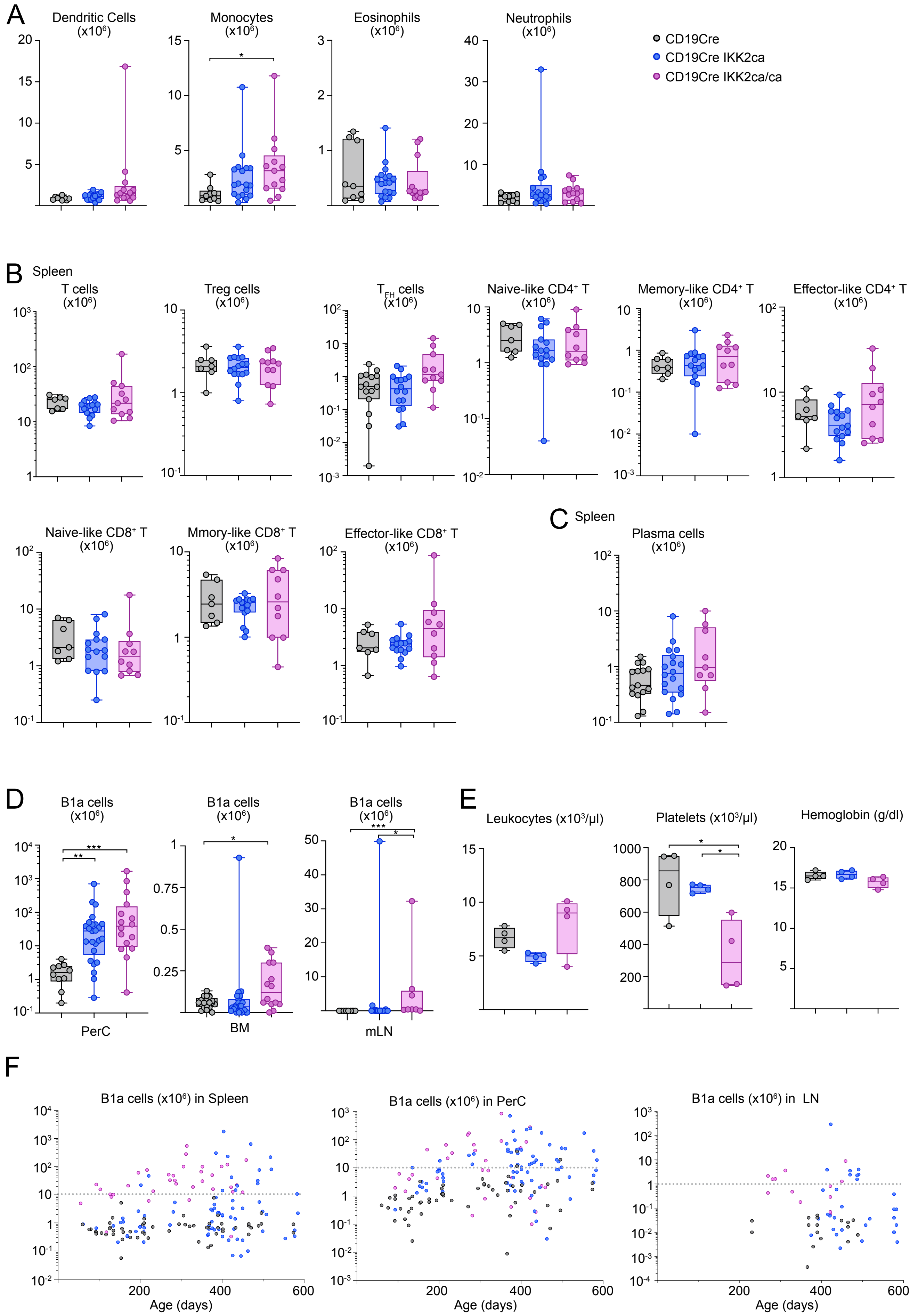

G

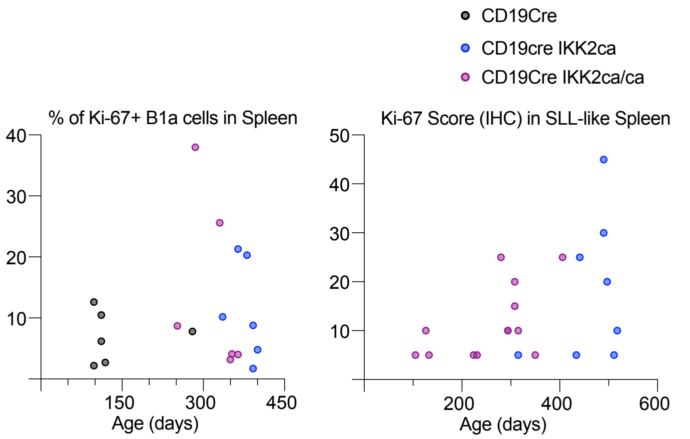

H

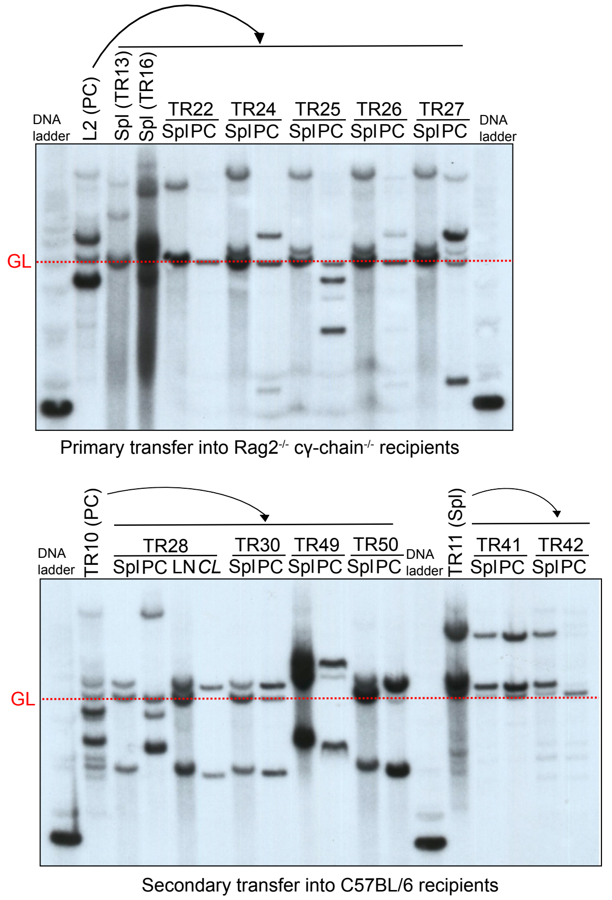

I

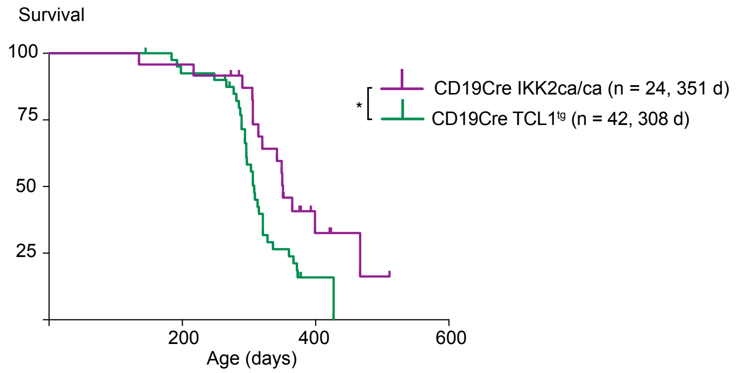

Suppl. Figure S2: Constitutive IKK2 activity induces lymphomagenesis in a dose-dependent manner

A-D) *Ex vivo* analysis of spleen, mesenteric lymph nodes (mLN), peritoneal cavity (PerC) and bone marrow (BM) of aged CD19Cre IKK2ca compound mice (6-12 months).

A) Absolute numbers of splenic myeloid subpopulations.

B) Absolute numbers of splenic T cells and T cell subpopulations.

C) Absolute numbers of splenic plasma cells.

D) Absolute numbers of B1a cells in peritoneal cavity (PerC), bone marrow (BM) and mesenteric lymph nodes (mLN).

E) Peripheral blood analysis of CD19Cre IKK2ca and CD19Cre IKK2ca/ca mice compared to CD19Cre control mice (7-11 months old) using the scil animal blood counter. Absolute numbers of leukocytes, platelets and hemoglobin levels are shown.

F) Cumulative *ex vivo* analysis of mice at different ages. Scatter plot showing absolute numbers of B1a cells in spleen, peritoneal cavity (PerC) and lymph nodes (LN).

G) *Ex vivo* analysis of splenic Ki-67 expression. Percentage of Ki-67$^+$ splenic B1a cells measured by flow cytometry and Ki-67 score in spleens assessed by immunohistochemistry (IHC). Data are shown for individual mice of the indicated genotypes, plotted according to age.

H) Southern blot analysis of StuI-digested DNA isolated from lymphomas arising in mice upon adoptive transfer of lymphoma cells of the indicated genotypes. The employed JH probe detects IgH VDJ rearrangements. Arrows indicate the transfer of primary lymphomas (L) to transferred lymphomas (TR) expanding in individual recipient mice or they show secondary transfers. GL: germline configuration.

I) Kaplan Meier survival curves comparing CD19Cre TCL1^{tg} (curve as shown in Fig. 3C) and CD19Cre IKK2ca/ca (as shown in Fig. 2C) mice. Median survival and number of mice are indicated.

* $p < 0.05$, ** $p \leq 0.01$, *** $p \leq 0.001$, **** $p \leq 0.0001$, one-way ANOVA followed by Tukey's multiple comparison test or Kruskal Wallis test followed by Dunn's multiple comparison test. For full statistical analysis see Table S2.

Neutrophils: CD11c$^-$ Mac1$^+$ SiglecF$^-$ F4/80$^-$ Gr1$^+$, Dendritic Cells: CD11c$^+$, monocytes: CD11c$^-$ Mac1$^+$ SiglecF$^-$ F4/80^{int} Gr1^{int}, Eosinophils: CD11c$^-$ Mac1$^+$ SiglecF$^+$ F4/80^{hi},

T cells: TCRβ$^+$, CD4$^+$ T cells: TCRβ$^+$ CD4$^+$ CD25$^-$ CD8$^-$, naive-like CD4$^+$ T cells: TCRβ$^+$ CD4$^+$ CD8$^-$ CD62L$^+$ CD44$^-$ CD25$^-$, memory-like CD4$^+$ T cells: TCRβ$^+$ CD4$^+$ CD8$^-$ CD62L$^+$ CD44$^+$ CD25$^-$, effector-like CD4$^+$ T cells: TCRβ$^+$ CD4$^+$ CD8$^-$ CD62L$^-$ CD44$^+$ CD25$^-$, regulatory T cells (Treg): TCRβ$^+$ CD4$^+$ CD8$^-$ CD25$^+$, CD8$^+$ T cells: TCRβ$^+$ CD8$^+$ CD4$^-$, naive-like CD8$^+$ T cells: TCRβ$^+$ CD4$^-$ CD8$^+$ CD62L$^+$ CD44$^-$, memory-like CD8$^+$ T cells: TCRβ$^+$ CD4$^-$ CD8$^+$ CD62L$^+$ CD44^{hi}, effector-like CD8$^+$ T cells: TCRβ$^+$ CD4$^-$ CD8$^+$ CD62L$^-$ CD44$^+$, follicular helper T cells (T_{FH}): TCRβ$^+$ CD4$^+$ CXCR5^{hi} PD1^{hi},

Plasma cells: CD138$^+$ CD19$^-$ (validated with IRF4 and Blimp1), B1a cells: CD19^{hi} B220^{low} CD5$^+$

Supplemental Figure S3

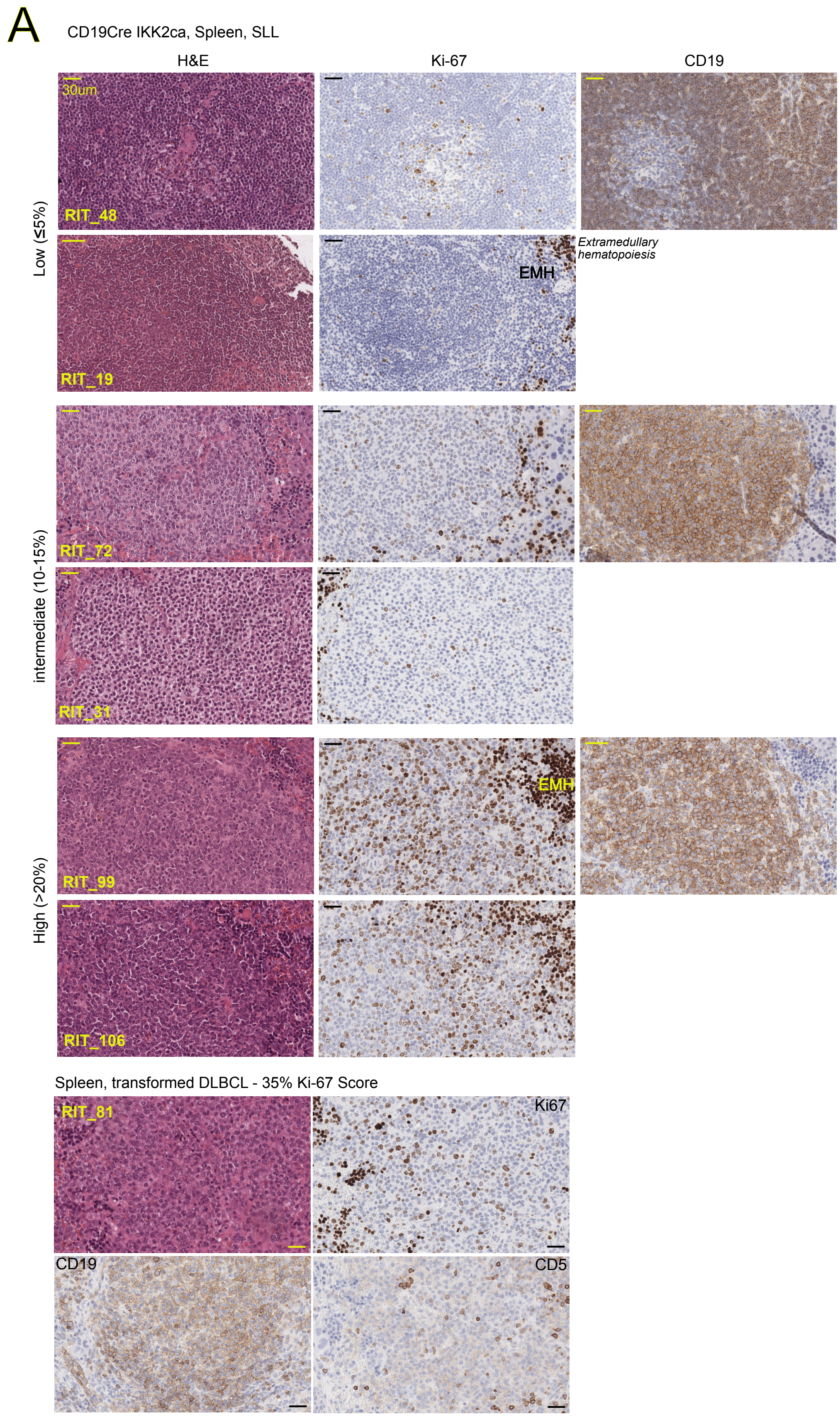

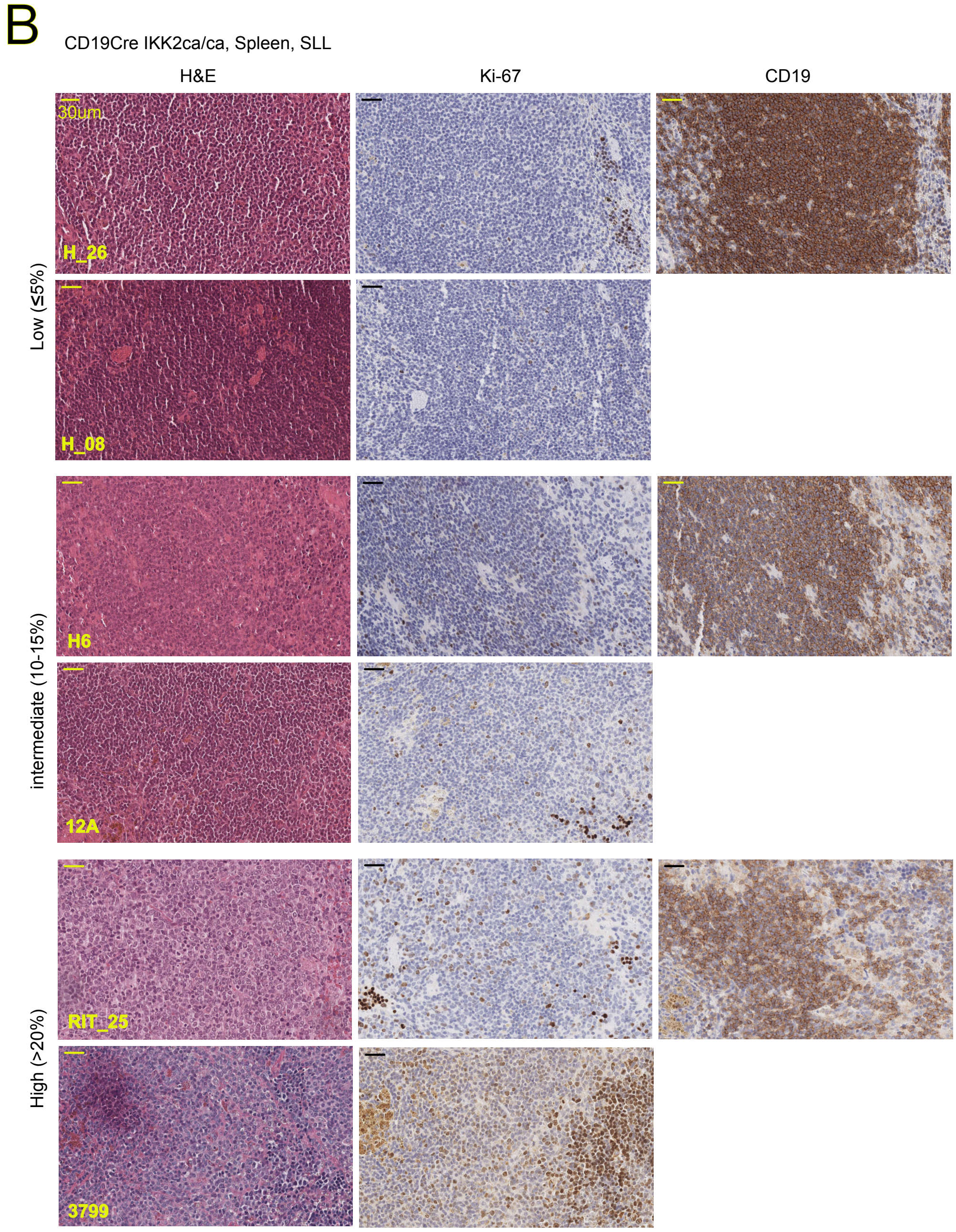

Suppl. Figure S3: Ki-67 staining of IKK2ca-driven lymphomas reveals variable proliferative activity

A-B) Representative H&E and Ki-67 immunohistochemistry (IHC) stainings of spleen sections are shown. Lymphomas depicted illustrate Ki-67 scores of ≤5%, 10–15%, and >20%. Sample IDs are indicated. Scale bars correspond to 30µm.

A) CD19Cre IKK2ca lymphoma spleen sections are shown for cases with small lymphocytic lymphoma (SLL)-like morphology and transformed diffuse large B cell lymphoma (DLBCL)-like features.

B) CD19Cre IKK2ca/ca lymphoma spleen sections are shown for cases with small lymphocytic lymphoma (SLL)-like morphology.

EMH: extramedullary hematopoiesis

Supplemental Figure S4

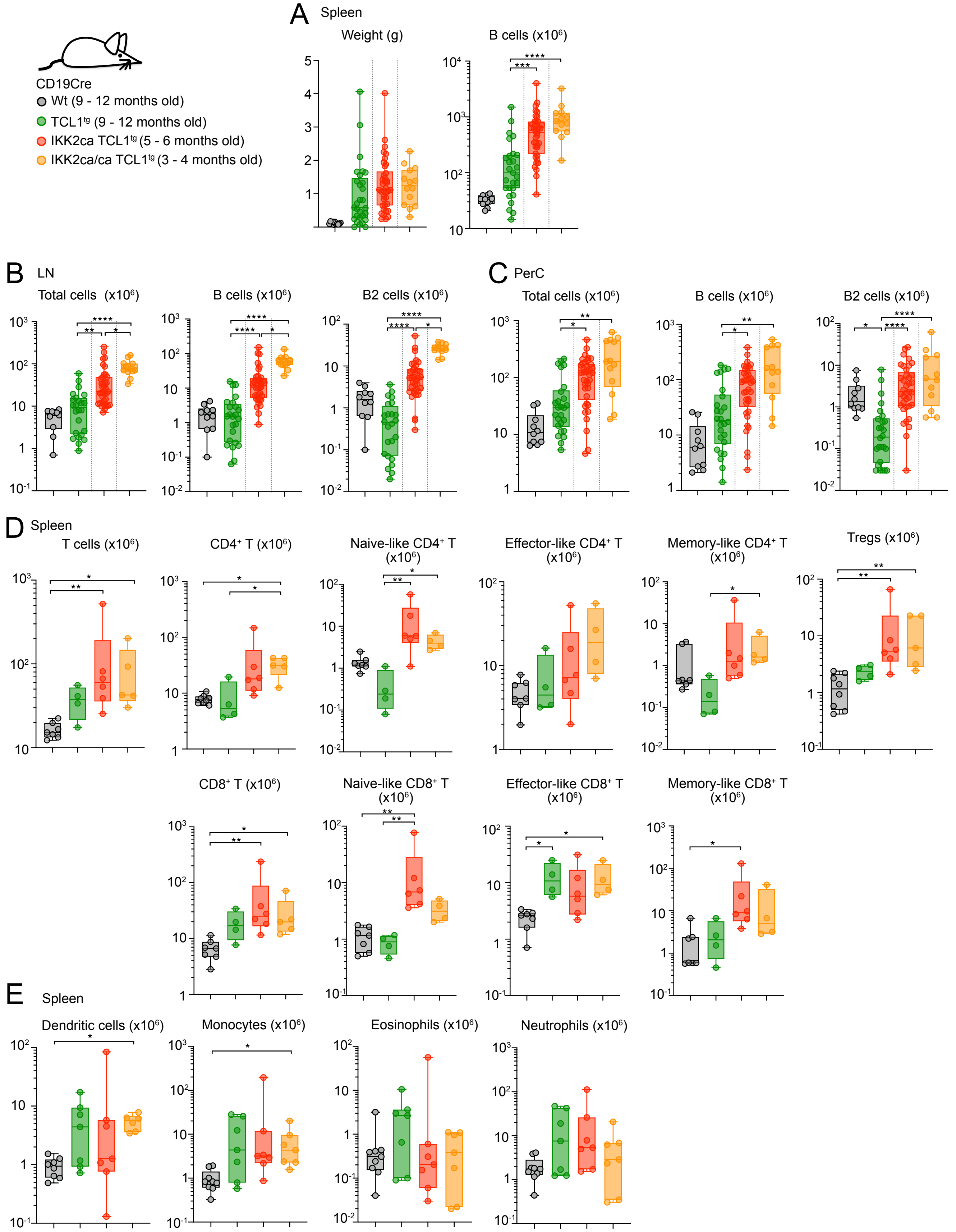

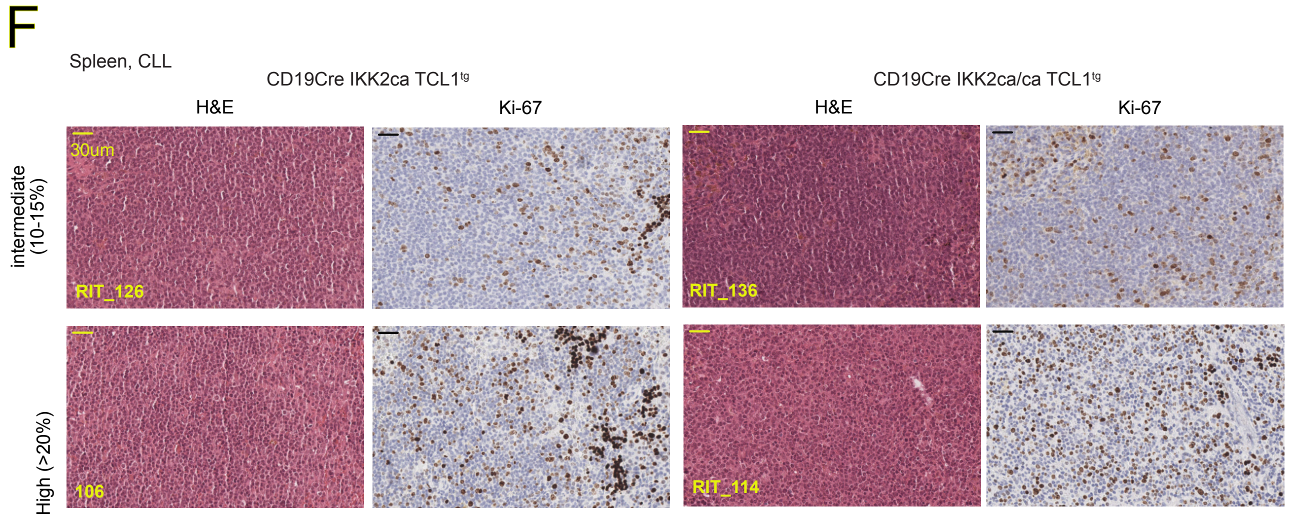

Suppl. Figure S4: B cell-intrinsic and -extrinsic consequences of synergistic effects of IKK2ca and TCL1 co-expression

A-E) *Ex vivo* analysis of spleen, lymph nodes (LN) and peritoneal cavity (PerC) of burdened mice: CD19Cre IKK2ca/ca TCL1^{tg} (3-4 months), CD19Cre IKK2ca TCL1^{tg} (5-6 months), CD19Cre TCL1^{tg} and CD19Cre control mice (9-12 months).

A) Spleen weight and absolute splenic B cell numbers.

B) Total cell numbers and absolute numbers of B cells and B2 cells in LNs.

C) Total cell number and absolute numbers of B cells and B2 cells in peritoneal cavity.

D) Absolute numbers of splenic T cells and T cell subpopulations.

E) Absolute numbers of dendritic cells, monocytes, eosinophils and neutrophils in the spleen.

F) Histological analysis of IKK2ca TCL1^{tg} and IKK2ca/ca TCL1^{tg} lymphomas showing variable percentages of Ki-67–positive cells. Representative H&E and immunohistochemistry (IHC) stainings of spleen sections are shown for cases with CLL-like morphology. Lymphomas are categorized based on Ki-67 positivity into intermediate (10-15%) or high (>20%) Ki-67 scores. Sample IDs are indicated. Scale bars correspond to 30µm.

* $p < 0.05$, ** $p \le 0.01$, *** $p \le 0.001$, **** $p \le 0.0001$, Kruskal Wallis test followed by Dunn's multiple comparison test . Comparisons to CD19Cre control mice are shown in selected cases only. For full statistical analysis see Table S2.

B cells: CD19$^+$, B2 cells: CD19$^+$ B220^{hi}

T-cells: TCRβ$^+$, CD4$^+$ T cells: TCRβ$^+$ CD4$^+$ CD25$^-$ CD8$^-$, naive-like CD4$^+$ T cells: TCRβ$^+$ CD4$^+$ CD8$^-$ CD62L$^+$ CD44$^-$ CD25$^-$, memory-like CD4$^+$ T cells: TCRβ$^+$ CD4$^+$ CD8$^-$ CD62L$^+$ CD44$^+$ CD25$^-$, effector-like CD4$^+$ T cells: TCRβ$^+$ CD4$^+$ CD8$^-$ CD62L$^-$ CD44$^+$ CD25$^-$, regulatory T cells (Treg): TCRβ$^+$ CD4$^+$ CD8$^-$ CD25$^+$, CD8$^+$ T cells: TCRβ$^+$ CD8$^+$ CD4$^-$, naive-like CD8$^+$ T cells: TCRβ$^+$ CD4$^-$ CD8$^+$ CD62L$^+$ CD44$^-$, memory-like CD8$^+$ T cells: TCRβ$^+$ CD4$^-$ CD8$^+$CD62L$^+$ CD44$^+$, effector-like CD8$^+$ T cells: TCRβ$^+$ CD4$^-$ CD8$^+$ CD62L$^-$ CD44$^+$

Neutrophils: CD11c$^-$ Mac1$^+$ SiglecF$^-$ F4/80$^-$ Gr1$^+$, Dendritic Cells: CD11c$^+$, Monocytes: CD11c$^-$ Mac1$^+$ SiglecF$^-$ F4/80^{int} Gr1^{int}, Eosinophils: CD11c$^-$ Mac1$^+$ SiglecF$^+$ F4/80^{hi}

Supplemental Figure S5

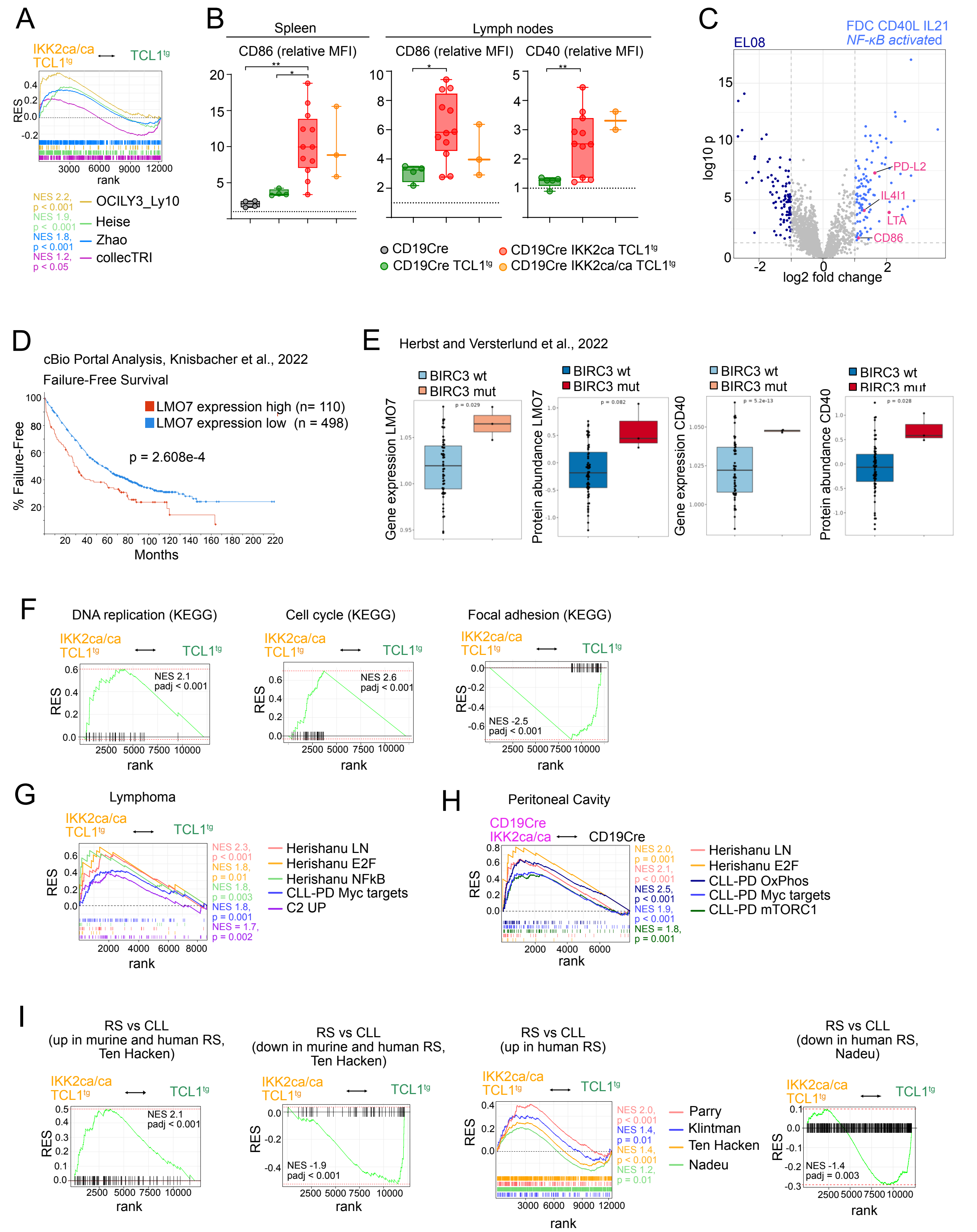

J

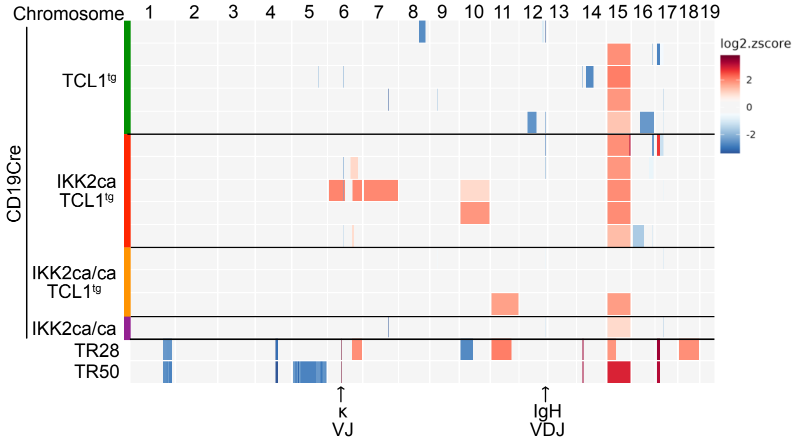

Suppl. Figure S5: Transcriptomic and chromosomal analysis of IKK2ca/ca- and TCL1-expressing lymphomas

A) Gene set enrichment analysis (GSEA) of different published NF-κB target gene signatures in IKK2ca/ca TCL1^{tg} lymphomas compared to TCL1^{tg} lymphomas.

B) CD40 and CD86 expression on B1a cells in spleen and lymph nodes of diseased IKK2ca/ca TCL1^{tg}, IKK2ca TCL1^{tg} and TCL1^{tg} mice as measured by flow cytometry. Median fluorescence intensity (MFI) was normalized to B2 cells of CD19Cre control mice.

C) Volcano plot showing differentially secreted proteins of primary CLL cells (n = 20) in co-culture with follicular dendritic cells overexpressing CD40L and IL21 (FDC CD40L IL21) compared to co-culture with EL08 stroma cells. Proteins that were differentially expressed in the secretome of feeder cells alone (FDC CD40L IL21 vs EL08) were excluded. The list of upregulated proteins is included in Table S2.

D) Failure-free survival of CLL patients with different LMO7 expression levels based on RNA sequencing data from Knisbacher et al.. Survival curve was generated with cBioPortal, groups were divided into high and low expressions using a z-score cutoff of 1[1–3].

E) Proteogenomic analysis of CLL patient samples showing gene expression and protein abundance of LMO7 and CD40 in BIRC3 mutant (mut) samples compared to BIRC3 wildtype (wt) samples. Data was obtained from dietrichlab.de/ShinyApps/CLL_Proteomics/[4].

F) GSEA of different KEGG pathway gene signatures in IKK2ca/ca TCL1^{tg} lymphomas compared to TCL1^{tg} lymphomas.

G) GSEA of gene signatures associated with proliferative and aggressive CLL in IKK2ca/ca TCL1^{tg} lymphomas compared to TCL1^{tg} lymphomas.

H) GSEA of gene signatures associated with proliferative and aggressive CLL in IKK2ca/ca expressing peritoneal B1a cells compared to CD19Cre controls.

I) GSEA of gene signatures that were described to be up- or downregulated in murine and/or human Richter syndrome (RS) vs CLL in IKK2ca/ca TCL1^{tg} lymphomas compared to TCL1^{tg} lymphomas.

J) Heatmap depicts chromosomal gains (red) and losses (blue) measured by comparative genomic hybridization (CGH) array analysis of splenic B1a tumors from indicated genotypes and the primary cell lines TR28 and TR50, derived from serial adoptive transfers of originally one primary IKK2ca/ca lymphoma (See Fig. S2H). Focal losses on chromosome 6 are related to the rearrangement of the kappa chain and focal losses including loss of Adam6a/b[5] on chromosome 12 are attributable to VDJ recombination.

RES = running enrichment score, NES = normalized enrichment score, rank = rank in gene list (ranked by stat)

* $p < 0.05$, ** $p \leq 0.01$, one-way ANOVA followed by Tukey's multiple comparison test Kruskal Wallis test followed by Dunn's multiple comparison test. Comparisons to CD19cre control mice are shown in selected cases only. For full statistical analysis see Table S2.

Gene signatures used for GSEA are listed in Table S3A,G or can be found in the KEGG pathway database; GSEA statistics are shown in Table S3C, F, H, I.

Supplemental Figure S6

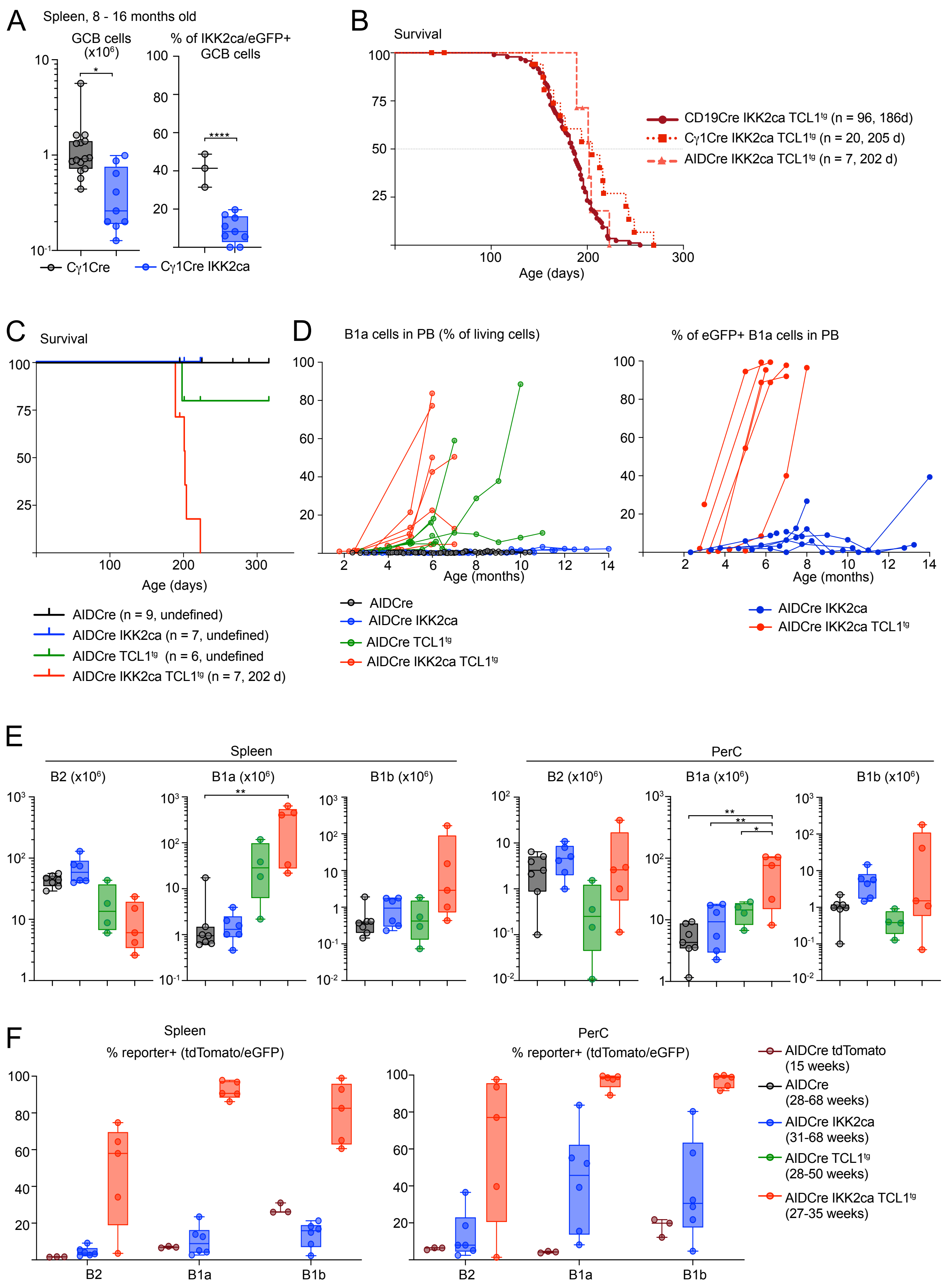

Suppl. Figure S6: Competitive advantage of B1a cells expressing IKK2ca, alone and in combination with TCL1

A) Total numbers of germinal center B cells (GCB) and recombined (eGFP$^+$) GCB in spleens of aged mice (8-16 months).

B) Kaplan Meier survival curves comparing genotypes CD19Cre IKK2ca TCL1^{tg} (curve as shown in Fig. 3C), Cγ1Cre IKK2ca TCL1^{tg} (as shown in Fig. 5A) and AIDCre IKK2ca TCL1^{tg}. The number of mice and median survival are indicated.

C) Kaplan-Meier survival curves for AIDCre-CLL cohort, number of mice and median survival are indicated. AIDCre IKK2ca TCL1^{tg} curve as shown in B.

D) The left scatter plot shows the percentage of B1a cells in peripheral blood (PB) over time in AIDCre mice of the indicated genotypes. The right scatter plot shows percentage of recombined (eGFP^{+}) B1a cells in peripheral blood over time. The lines connect analyses at different timepoints for individual mice.

E) Total numbers of B2, B1a and B1b cells in spleen and peritoneal cavity (PerC) of aged mice (6 - 16 months).

F) Percentage of recombined cells (eGFP^{+} for AIDCre IKK2ca and AIDCre IKK2ca TCL1^{tg}, tdTomato^{+} for AIDCre tdTomato) per B cell subpopulations in spleen and peritoneal cavity measured by flow cytometry. Due to the shorter loxP-flanked STOP cassette, the LSL-tdTomato(0.9 kb STOP cassette) allele is more sensitive reporter of Cre activity compared to LSL-IKK2ca[6] and LSL-CAR[7] (both 2.5 kb STOP cassette)[8]. Therefore, the proportion of tdTomato expressing cells in the B cell subsets shown in this figure is most likely higher than the proportion of unselected IKK2ca-expressing cells would be.

* $p < 0.05$, ** $p \leq 0.01$, *** $p \leq 0.001$, **** $p \leq 0.0001$, one-way ANOVA followed by Tukey's multiple comparison test or Kruskal Wallis test followed by Dunn's multiple comparison test.

Full statistics are shown in Table S2.

Supplemental Figure S7

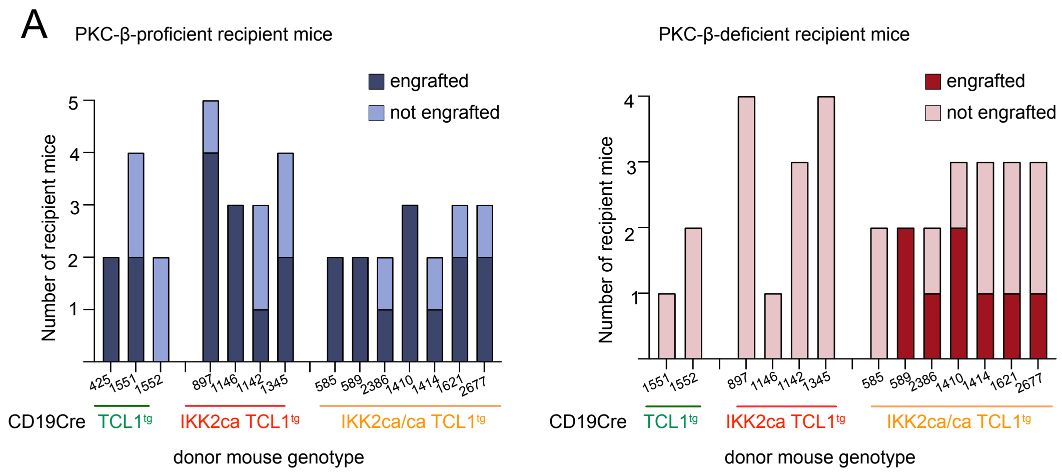

Suppl. Figure S7: IKK2ca/ca-expressing TCL1^{tg} CLL cells engraft in PKC-β-deficient mice

A) Overview of all transplanted mice showing engraftment of lymphomas from different donor mice in PKC-β-proficient and deficient recipient mice.

References:

1. Bruijn, I. de et al. Analysis and Visualization of Longitudinal Genomic and Clinical Data from the AACR Project GENIE Biopharma Collaborative in cBioPortal. Cancer Res. 83, 3861–3867 (2023).

2. Gao, J. et al. Integrative Analysis of Complex Cancer Genomics and Clinical Profiles Using the cBioPortal. Sci. Signal. 6, pl1 (2013).

3. Cerami, E. et al. The cBio Cancer Genomics Portal: An Open Platform for Exploring Multidimensional Cancer Genomics Data. Cancer Discov. 2, 401–404 (2012).

4. Herbst, S. A. et al. Proteogenomics refines the molecular classification of chronic lymphocytic leukemia. Nat. Commun. 13, 6226 (2022).

5. Featherstone, K., Wood, A. L., Bowen, A. J. & Corcoran, A. E. The Mouse Immunoglobulin Heavy Chain V-D Intergenic Sequence Contains Insulators That May Regulate Ordered V(D)J Recombination. J. Biol. Chem. 285, 9327–9338 (2010).

6. Sasaki, Y. et al. Canonical NF-kappaB activity, dispensable for B cell development, replaces BAFF-receptor signals and promotes B cell proliferation upon activation. Immunity 24, 729–739 (2006).

7. Heger, K. et al. A novel Cre recombinase reporter mouse strain facilitates selective and efficient infection of primary immune cells with adenoviral vectors. European journal of immunology 45, 1614–1620 (2015).

8. Bedolla, A. M. et al. A comparative evaluation of the strengths and potential caveats of the microglial inducible CreER mouse models. Cell Rep. 43, 113660–113660 (2024).
