# Supplementary material for: Strong constitutive NF-κB signaling in B cells drives SLL/CLL-like lymphomagenesis and overcomes microenvironmental dependencies
Source: Leukemia. 2026 Jan 16;40(3):522–39. doi: 10.1038/s41375-025-02844-8 (PMC12960208; doi:10.1038/s41375-025-02844-8)
Supplement: Supplementary file 3 — Supplemental Table 1 Resources [file 41375_2025_2844_MOESM3_ESM.docx]

**Supplementary Table S1A.** PCR primers used for genotyping mouse strains

| **Allele** | **Sequence (5’-3’ orientation)** | **Amplicon size** |
| --- | --- | --- |
| AIDCre | CACTCGTTGCATCGACCGGTAATG GGACCCAACCCAGGAGGCAGATGT CCTCTAAGGCTTCGCTGTTATTACCAC | Wild type allele: 484 bp Knock-in allele: 283 bp |
| CD19Cre | CCCAGAAATGCCAGATTA  AACCAGTCAACACCCTTCC  CCAGACTAGATACAGACCAG | Wild type allele: 452 bp  Knock-in allele: 500 bp |
| Cγ1Cre | TGTTGGGACAAACGAGCAATC  GGTGGCTGGACCAATGTAAATA  GTCATGGCAATGCCAAGGTCGCTAG | Wild type allele: 250 bp  Knock-in allele: 470 bp |
| Rosa26-Knock-in for the IKK2ca allele | AAAGTCGCTCTGAGTTGTTATC  GATATGAAGTACTGGGCTCTT  GCATCGCCTTCTATCGCCT | Wild type allele: 570 bp  Knock-in allele: 450 bp |
| Rosa26-CAR | CCTGCTGTGCTTCGTGCTCC  CGTAACATCTCGCACCTGAAGGC | Knock-in allele: 435 bp |
| Eµ-TCL1tg | AGTGGTAAATATAGGGTTGTCTACACG  CCCGTAACTGTAACCTATCCTTTA | Transgene allele: 250 bp |
| PKC-ß KO | CAGGGTCGAATTGCCATCCTCCA  CTTGGGTGGAGAGGCTATTC  AGCCACTCTCGGTGCTGTG | Wild type allele: 391 bp  Knock out allele: 800 bp |

**Supplementary Table S1B.** PCR primers used for amplification of the Immunoglobulin Heavy Chain (IgH) VDJ rearrangement

| **Primer name** | **Sequence (5´-3’ orientation)** | **Amplicon size** |
| --- | --- | --- |
| MsVhE AH | TCGAGTTTTTCAGCAAGATGAGGTGCAGCTGCAGGAGTCTGG |  |
| JH1/4 Arnaout | CTTACCTGAGGAGACGGTGAC | 350 bp |
| JH2/3 Arnaout | AGGACTCACCTGAGGAGAC | 350 bp |
| JH2 Arnaout | AGGACTCACCTGCAGAGAC | 350 bp |
| JH4rev | CTGAGGAGACGGTGACTGAGG | JH1 1.6kb  JH2 1.3kb  JH3 1.0kb |

**Supplementary Table S1C.** PCR primers used for RT-PCR and amplification of the IgM BCR of lymphomas

| **Primer name** | **Application** | **Sequence** |
| --- | --- | --- |
| **First-strand cDNA synthesis** | | |
| SmartNNNext | 5′ – template-switch oligo with sequencing illumina adapter. U = dU, rG-riboG | AGATGUGTAUAAGAGACAGNNNNUNNNNUNNNNUCTT(rG)_4_ |
| **Mouse IGH cDNA synthesis primer mix** | | |
| mIGM_r1 | Primer for cDNA synthesis, mouse IgM heavy-chain mRNA | CTGGATGACTTCAGTGTTGT |
| **Mouse IGL cDNA synthesis primer mix** | | |
| mIGLC_r1 | Primer for cDNA synthesis, mouse IgL light-chain mRNA | TGTACCATYTGCCTTCCAG |
| mIGKC_r1 | Primer for cDNA synthesis, mouse IgK light-chain mRNA | ACTGCCATCAATCTTCCAC |
| **First PCR amplification** | | |
| Common primer | Step-out primer, anneals on the switch adaptor | AGATGTGTATAAGAGACAG |
| **Mouse IGH reverse primer mix** | | |
| Common-mIGG12_r2 | Nested primer with sequencing illumina adaptor, mouse IgG1/IgG2 heavy-chain cDNA | AGATGTGTATAAGAGACAGAGTGGATAGACMGATG |
| Common-mIGG3_r2 | Nested primer with sequencing illumina adaptor, mouse IgG3 heavy-chain cDNA | AGATGTGTATAAGAGACAGAAGGGATAGACAGATG |
| Common-mIGA_r2 | Nested primer with sequencing illumina adaptor, mouse IgA heavy-chain cDNA | AGATGTGTATAAGAGACAGTCAGTGGGTAGATGGTG |
| Common-mIGM_r2 | Nested primer with sequencing illumina adaptor, mouse IgM heavy-chain cDNA | AGATGTGTATAAGAGACAGGGGGGAAGACATTTGG |
| Common-mIGD_r2 | Nested primer with sequencing illumina adaptor, mouse IgD heavy-chain cDNA | AGATGTGTATAAGAGACAGCTCTGAGAGGAGGAAC |
| Common-mIGE_r2 | Nested primer with sequencing illumina adaptor, mouse IgE heavy-chain cDNA | AGATGTGTATAAGAGACAGAAGGGGTAGAGCTGAG |
| **Mouse IGL reverse primer mix** | | |
| Common-mIGL_r2 | Nested primer with sequencing illumina adaptor, mouse IgL heavy-chain cDNA | AGATGTGTATAAGAGACAGAGRGGAAGGTGGAAAC |
| Common-mIGK_r2 | Nested primer with sequencing illumina adaptor, mouse IgK heavy-chain cDNA | AGATGTGTATAAGAGACAGGGATGGTGGGAAGATG |
| **Second PCR amplification** | | |
| F-common | Step-out primer with sequencing and P7 illumina adapters | TCGTCGGCAGCGTCAGATGTGTATAAGAGACAG |
| R- common | Step-out primer with sequencing and P5 illumina adapters | GTCTCGTGGGCTCGGAGATGTGTATAAGAGACAG |
| **Third PCR amplification** | | |
| Fc_i7^1^ | Step-out primer with index 1 illumina adapter | CAAGCAGAAGACGGCATACGAGAT[i7]GTCTCGTGGGCTCGG |
| Fc_i5^2^ | Step-out primer with index 2 illumina adapter | AATGATACGGCGACCACCGAGATCTACAC[i5]TCGTCGGCAGCGTC |

^1, 2^ - illumina Nextera index adapters (i5 and i7). See illumina Nextera DNA library preparation reference guide and illumina adapters sequences list for more information.

**Supplementary Table S1D**. Flow cytometry antibodies

| **Antibody** | **Clone** | **Company** | **RRID** |
| --- | --- | --- | --- |
| B220 | RA3-6B2 | BioLegend | AB_312988, AB_893354, AB_2561394 |
| CD138 | 281-2 | BD  BioLegend | AB_394999  AB_10916119, AB_11204257 |
| CD19 | MB19-1 | eBioscience | AB_469358 |
| CD1d | 1B1 | eBioscience | AB_465483, AB_466308 |
| CD16/32 (Fc block) | 93 | eBioscience | AB_467132 |
| CD21/CD35 | eBio8D9 | eBioscience | AB_466390, AB_465588 |
| CD23 | B3B4 | eBioscience | AB_469604 |
| CD25 | PC61.5 | eBioscience | AB_10671550, AB_469366 |
| CD3ε | 145-2C11 | BD BioLegend | AB_394593  AB_312676 |
| CD38 | 90 | BioLegend | AB_312932 |
| CD4 | RM4-5 | BioLegend | AB_893325 |
| CD44 | IM7 | eBioscience | AB_469623 |
| CD5 | 53-7.3 | eBioscience | AB_1603250, AB_466339, AB_2539168 |
| CD62L (L-Selectin) | MEL-14 | eBioscience  BioLegend | AB_465721  AB_2561537 |
| CD8-alpha | 53-6.7 | eBioscience  BioLegend | AB_11155594  AB_493702 |
| CD95 | Jo2 | BD | AB_395330 |
| Ly-6G/Ly6C (Gr-1) | RB6-8C5 | BioLegend  ThermoFisher | AB_313368  AB_2621610 |
| Ly-6G | 1A8 | BioLegend | AB_1186105 |
| IgD | 11-26c/11-26  11-26c.2a | eBioscience  BioLegend | AB_2573821  AB_466860, AB_1575115 |
| IgM | II/41 | eBioscience | AB_469655, AB_466675 |
| IRF4 | 3E4 | eBioscience | AB_2574135, AB_2573912,  AB_10852721 |
| CD11b (Mac-1) | M1/70 | BioLegends  eBioscience | AB_2561390  AB_466359, AB_1582236 |
| CD11c | N418 | eBioscience | AB_469346 |
| CXCR5 | 2G8 | BD | AB_394301, AB_394300 |
| CXCR4 | 2B11/CXCR4 | BD | AB_394305 |
| F4/80 | CI:A3-1 | BioRad, old AbD serotec | AB_322046 |
| PD-1 | J43 | eBioscience | AB_10853805 |
| Siglec-F | E50-2440  S17007L | BD  BioLegen | AB_394341  AB_2814066 |
| TCR beta | H57-597 | eBioscience | AB_1272173, AB_2802349 |
| CAR | E1-1 | Santa Cruz | AB_783493 (discontinued) |
| Human TCL1 | eBio1-21 | eBioscience | AB_11149309, AB_10853340 |
| Human TCL1 | 27D6/20 | MBL | AB_1279328 (discontinued) |
| Phospho NFkB p65 (RelA) | 93H1 | Cell Signaling | AB_10706937 |
| IkBa | L3A5 | Cell Signaling | AB_2797687 |
| Ki67 | SolA15 | eBioscience | AB_11040981 |

**Supplementary Table S1E.** Western Blot antibodies

| **Antibody** | **Clone** | **Catalog number** | **Company** | **Dilution** | **RRID** |
| --- | --- | --- | --- | --- | --- |
| IKK2 | L570 | 2678 | Cell Signaling Technology | 1:1000 | AB_2122301 |
| IkBa | L35A5 | 4814 | Cell Signaling Technology | 1:1000 | AB_390781 |
| p100 | polyclonal | 4882 | Cell Signaling Technology | 1:1000 | AB_10695537 |
| RelB | C1E4 | 4922 | Cell Signaling Technology | 1:1000 | AB_2179173 |
| IkBe | M-364 | sc7155 | Santa Cruz | 1:1000 | AB_2235977 |
| Actin | C4 | sc-47778 | Santa Cruz | 1:10000 | AB_626632  AB_2714189 |
| HRP-conjugated anti-rabbit |  | 711-035-152 | Jackson ImmunoResearch | 1:10000 | AB_10015282 |
| HRP-conjugated anti-mouse |  | 715-035-150 | Jackson ImmunoResearch | 1:10000 | AB_2340770 |
